# Plasmodium falciparum and Helminth Coinfections Increase IgE and Parasite-Specific IgG Responses

**DOI:** 10.1128/Spectrum.01109-21

**Published:** 2021-12-08

**Authors:** Rebeca Santano, Rocío Rubio, Berta Grau-Pujol, Valdemiro Escola, Osvaldo Muchisse, Inocência Cuamba, Marta Vidal, Pau Cisteró, Gemma Ruiz-Olalla, Ruth Aguilar, Maria Demontis, Jose Carlos Jamine, Anélsio Cossa, Charfudin Sacoor, Jorge Cano, Luis Izquierdo, Chetan E. Chitnis, Ross L. Coppel, Virander Chauhan, David Cavanagh, Sheetij Dutta, Evelina Angov, Deepak Gaur, Lisette van Lieshout, Bin Zhan, Jose Muñoz, Gemma Moncunill, Carlota Dobaño

**Affiliations:** a Malaria Immunology Group, ISGlobal, Hospital Clínic - Universitat de Barcelona, Barcelona, Spain; b Centro de Investigação em Saúde de Manhiça (CISM), Maputo, Mozambique; c Fundación Mundo Sano, Buenos Aires, Argentina; d Department of Parasitology, Centre of Infectious Diseases, Leiden University Medical Centre (LUMC), Leiden, the Netherlands; e Communicable and Non-communicable Diseases Cluster (UCN), WHO Regional Office for Africa, Brazzaville, Republic of Congo; f Malaria Parasite Biology and Vaccines, Department of Parasites & Insect Vectors, Institut Pasteur, Paris, France; g Department of Microbiology, Faculty of Medicine, Nursing and Health Sciences, Monash University, Melbourne, Australia; h Malaria Group, International Centre for Genetic Engineering and Biotechnology (ICGEB), New Delhi, India; i Institute of Immunology and Infection Research, University of Edinburgh, Edinburgh, UK; j Walter Reed Army Institute of Research (WRAIR), Silver Spring, Maryland, USA; k Laboratory of Malaria & Vaccine Research, School of Biotechnology, Jawaharlal Nehru University, New Delhi, India; l Baylor College of Medicine (BCM), Houston, Texas, USA; m CIBER de Enfermedades Infecciosas, Madrid, Spain; Weill Cornell Medicine

**Keywords:** antibodies, coinfection, helminths, IgE, IgG, luminex, malaria

## Abstract

Coinfection with Plasmodium falciparum and helminths may impact the immune response to these parasites because they induce different immune profiles. We studied the effects of coinfections on the antibody profile in a cohort of 715 Mozambican children and adults using the Luminex technology with a panel of 16 antigens from P. falciparum and 11 antigens from helminths (*Ascaris lumbricoides*, hookworm, Trichuris trichiura, Strongyloides stercoralis, and *Schistosoma* spp.) and measured antigen-specific IgG and total IgE responses. We compared the antibody profile between groups defined by P. falciparum and helminth previous exposure (based on serology) and/or current infection (determined by microscopy and/or qPCR). In multivariable regression models adjusted by demographic, socioeconomic, water, and sanitation variables, individuals exposed/infected with P. falciparum and helminths had significantly higher total IgE and antigen-specific IgG levels, magnitude (sum of all levels) and breadth of response to both types of parasites compared to individuals exposed/infected with only one type of parasite (*P* ≤ 0.05). There was a positive association between exposure/infection with P. falciparum and exposure/infection with helminths or the number of helminth species, and *vice versa* (*P* ≤ 0.001). In addition, children coexposed/coinfected tended (*P* = 0.062) to have higher P. falciparum parasitemia than those single exposed/infected. Our results suggest that an increase in the antibody responses in coexposed/coinfected individuals may reflect higher exposure and be due to a more permissive immune environment to infection in the host.

**IMPORTANCE** Coinfection with Plasmodium falciparum and helminths may impact the immune response to these parasites because they induce different immune profiles. We compared the antibody profile between groups of Mozambican individuals defined by P. falciparum and helminth previous exposure and/or current infection. Our results show a significant increase in antibody responses in individuals coexposed/coinfected with P. falciparum and helminths in comparison with individuals exposed/infected with only one of these parasites, and suggest that this increase is due to a more permissive immune environment to infection in the host. Importantly, this study takes previous exposure into account, which is particularly relevant in endemic areas where continuous infections imprint and shape the immune system. Deciphering the implications of coinfections deserves attention because accounting for the real interactions that occur in nature could improve the design of integrated disease control strategies.

## INTRODUCTION

Malaria and helminthiasis are endemic parasitic diseases in tropical and subtropical areas, especially in impoverished countries with poor water and sanitation access. Their overlapping spatial distribution makes coinfections with these pathogens a frequent event (1). Both cause a high burden of morbidity, and malaria is a leading cause of mortality, particularly in children (2–4). The highest burden of malaria cases (82%) and deaths (94%) occurs in sub-Saharan Africa (2), where Plasmodium falciparum is the most prevalent species causing malaria (5). Helminths such as *Schistosoma* spp. and soil-transmitted helminths (STH) are also prevalent in Sub-Saharan Africa (3, 4).

P. falciparum and helminths induce different types of immune responses. On one hand, the clearance of P. falciparum infection is achieved by an initial T helper (Th)1 response with the production of IgG antibodies, mainly from the cytophilic IgG1 and IgG3 subclasses (6, 7). On the other hand, helminths generally induce a Th2 polarization with the production of IgE and IgG4 antibodies (8–10). However, helminths are a heterogeneous group of parasites, each with a complex life cycle; therefore, differences exist by species and life cycle stage. Adding another layer of complexity, helminths are well known for their immunomodulating effects that deviate the cellular response toward a regulatory profile. This allows helminths to survive in the host for years and this may affect not only helminth antigens but also bystander antigens (8–11). Therefore, by altering the immunological balance, coinfections can impact immunity and course of infections.

However, there is scarce literature on the subject with contradictory results. Helminths have been associated with detrimental effects on P. falciparum infection, parasite load, and complications in some cases (12–21), with protective effects in other cases (22–31), or no effect (32). The outcome may be critically influenced by the species, timing (33), parasite load (23, 25, 31), and endpoint analyzed (infection or disease) (34). Regarding the effect of P. falciparum on helminth infections in humans, P. falciparum could also affect the frequency and course of helminth infections, perhaps by delaying or dampening the production of required Th2 cytokines (35–37). In addition, the concept of immunological memory suggests that previous exposures shape the present immune function and therefore, cumulative past infections could define an individuals’ basal immune state (38, 39). For this reason, taking into account prior exposure to pathogens is crucial to decipher the implications of coinfections in the immune response.

Based on the different immune responses triggered by P. falciparum and helminths and the immunomodulating effect of helminths, we hypothesized that prior exposure to P. falciparum and helminths or their coinfection would have an impact on the immune response to these parasites. Thus, our objective was to describe the effects of P. falciparum and helminth coexposure and coinfections on the antibody profiles in an endemic cohort from Southern Mozambique. We measured antigen-specific IgG and total IgE responses with a multiplex suspension array technology (Luminex) including a panel of 16 antigens from different life stages of P. falciparum and 11 antigens from helminths (STH and *Schistosoma* spp.). The antibody profile was compared between different exposure/infection groups defined by previous exposure (based on serology) and/or the presence of current infection (determined by microscopy and/or qPCR).

## RESULTS

### Characteristics of the study population.

The study included 715 subjects (363 children and 352 adults) from six different locations of Manhiça district, in the Maputo province, Southern Mozambique (Table 1). From all the participants, 9.93% were asymptomatically infected with P. falciparum as diagnosed by qPCR, and 53.57% carried a helminth infection (diagnosed by either qPCR and/or microscopy) (Table S1). Twenty-eight percent were infected by hookworm (Ancylostoma duodenale and/or Necator americanus), 14.41% by Trichuris trichiura, 12.03% by Schistosoma spp., 10.35% by Strongyloides stercoralis, and 7.41% by Ascaris lumbricoides. From those infected with helminths, 29.24% were infected with more than one helminth species, with the coinfection of hookworm with S. stercoralis being the most common (Table S2 and Fig. S1A). Assessed by serology, 91.05% of individuals had been exposed to P. falciparum and 79.16% to any helminth (69.51% to hookworm, 52.73% to T. trichiura, 43.92% to Schistosoma spp. 27.27% to S. stercoralis, and 20.70% to A. lumbricoides) (Table S1). Most of the helminth exposed individuals (77.21%) had also been exposed to multiple helminth species, mainly to hookworm and T. trichiura (Table S2 and Fig. S1B).

**TABLE 1:** Demographic, socioeconomic, water, and sanitation characteristics of the study population by exposure/infection group^*a*^

	MAL− HEL− (*N* = 21, 2.94%)	MAL− HEL+ (*N* = 43, 6.01%)	MAL+ HEL− (*N* = 67, 9.37%)	MAL+ HEL+ (*N* = 584, 82.68%)	*p*-value	Groups with significant differences
Sex					0.561	None
Female (*N* = 404)	9 (42.9%)	23 (53.5%)	40 (59.7%)	332 (56.8%)		
Male (*N* = 311)	12 (57.1%)	20 (46.5%)	27 (40.3%)	252 (43.2%)		
Age (yrs)	7.7 [6.0;11.5]	8.6 [6.7;9.9]	9.4 [6.9;11.8]	23.9 [10.7;50.8]	≤0.001	MAL− HEL− vs MAL+ HEL+, MAL− HEL+ vs MAL+ HEL+, MAL+ HEL− vs MAL+ HEL+
Age group					≤0.001	MAL− HEL− vs MAL+ HEL+, MAL− HEL+ vs MAL+ HEL+, MAL+ HEL− vs MAL+ HEL+
Children (*N* = 363)	21 (100.0%)	42 (97.7%)	62 (92.5%)	238 (40.8%)		
Adults (*N* = 352)	0 (0.0%)	1 (2.3%)	5 (7.5%)	346 (59.2%)		
Malaria % in location					0.397	None
High (*N* = 222)	5 (23.8%)	10 (23.3%)	25 (37.3%)	182 (31.2%)		
Low (*N* = 493)	16 (76.2%)	33 (76.7%)	42 (62.7%)	402 (68.8%)		
Helminth % in location					0.377	None
High (*N* = 445)	10 (47.6%)	27 (62.8%)	38 (56.7%)	370 (63.4%)		
Low (*N* = 270)	11 (52.4%)	16 (37.2%)	29 (43.3%)	214 (36.6%)		
Socioeconomic score	−0.16 [–0.22; –0.04]	−0.18 [–0.26; –0.08]	−0.14 [−0.26;−0.05]	−0.21 [−0.29; −0.10]	0.011	None
Ownership of latrine					0.21	None
No (*N* = 328)	10 (47.6%)	18 (41.9%)	23 (34.3%)	277 (47.5%)		
Yes (*N* = 386)	11 (52.4%)	25 (58.1%)	44 (65.7%)	306 (52.5%)		
Piped water access					0.339	None
Outside (*N* = 535)	13 (61.9%)	31 (72.1%)	47 (70.1%)	444 (76.2%)		
Inside (*N* = 179)	8 (38.1%)	12 (27.9%)	20 (29.9%)	139 (23.8%)		
Payment for water					0.813	None
No (*N* = 472)	12 (57.1%)	29 (67.4%)	43 (64.2%)	388 (66.6%)		
Yes (*N* = 242)	9 (42.9%)	14 (32.6%)	24 (35.8%)	195 (33.4%)		

a Comparisons were made between study groups, no exposure/infection (MAL− HEL−); only exposure/infection with helminths (MAL− HEL+); only exposure/infection with P. falciparum (MAL+ HEL−) or coexposure/coinfection with P. falciparum and helminths (MAL+ HEL+). Malaria and helminth percentage of infection in each location of the study were calculated and participants were classified into groups of locations with higher or lower percentage of infection compared to the overall median by type of parasite. Total sample size was *N* = 715, except for the socioeconomic score, ownership of latrine, piped water access, and payment for water that was *N* = 714. Data are provided as numbers and proportions except for age and socioeconomic score, where median and interquartile range are indicated. Proportions in each group column for each variable were calculated from the total number of each group indicated in the top row. Additionally, in the first column, total numbers for the whole study population are provided. The proportions for each group at the top row were calculated from the total sample size of the population.

For subsequent analyses, we considered only helminth infection regardless of the species. Combining the diagnosis of exposure and/or infections, 91.05% and 87.69% of individuals had been exposed to and/or were infected with P. falciparum and helminths, respectively (Table S1), with the following breakdown into exposure/infection groups: no exposure/infection (MAL− HEL− [*N* = 21, 2.94%]), single exposure/infection (MAL−HEL+ [*N* = 43, 6.01%] or MAL+ HEL− [*N* = 67, 9.37%]) and coexposure/coinfection (MAL+ HEL+ [*N* = 584, 82.68%]) (Table 1). The median antibody levels for each group, defined by exposure/infection, followed a similar pattern but were lower compared to the same groups defined by current infection only (Fig. S2). Using a combination of past exposure and current infection, we were able to capture a large proportion of exposed individuals not detected by current infection diagnosis (Table S3).

Females (*N* = 404) and males (*N* = 311) were equally distributed across exposure/infection study groups but there were significant differences in terms of age (Table 1). As expected, the MAL− HEL− group had the lowest age median (7.7 years), while the MAL+ HEL+ group had the highest age median (23.9 years) (Table 1). Further, the breakdown by age group revealed a very unbalanced distribution of children (5 to 14 years old) and adults (≥15 years old) across study groups, with the vast majority of adults in the MAL+ HEL+ group (Table 1).

The percentage of individuals with asymptomatic malaria and infection by any helminth was calculated for each of the six locations. Then, these were classified into “high” and “low” prevalence locations based on higher or lower percentage of infected individuals compared to the median percentage of infections from all locations (Table 1 and Table S4). No statistically significant differences were found by high or low location between study groups (Table 1).

Regarding socioeconomic, water, and sanitation metrics, such as socioeconomic score (SES), ownership of latrine, accessibility to piped water inside the household and payment for water, there were no statistically significant differences between exposure/infection groups (Table 1).

### Association of demographic, SES, water and sanitation variables with antibody levels.

**FIG 1:**
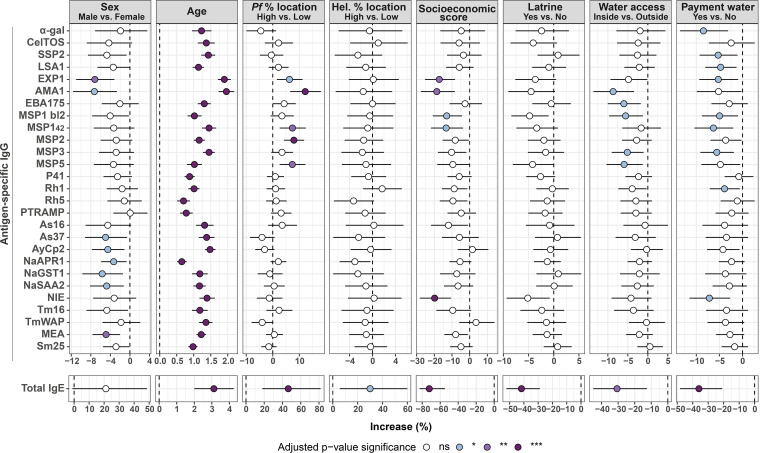
Association of demographic, socioeconomic, water, and sanitation metrics with antibody levels in univariable linear regression models. Forest plots show the association of sex, age, percentage of P. falciparum-infected individuals by location, percentage of helminth-infected individuals by location, socioeconomic score, owning a latrine, piped water accessibility, and payment for water with specific IgG levels to P. falciparum and helminth antigens and total IgE levels. Univariable linear regression models were fitted to calculate the estimates (dots) and 95% confidence intervals (CI) (lines). The represented values are the transformed betas and CI showing the percentage increase for each 10% increase in the case of age (log-log model) or in comparison with the reference category for the rest of the variables (log-linear models) (see “Statistical analysis” section for more details). The color of the dots represents the *P value* significance after adjustment for multiple testing by Benjamini-Hochberg, where ns, not significant and *, *P value* ≤ 0.05; **, *P value* ≤ 0.01; ***, *P value* ≤ 0.001. Pf, P. falciparum; Hel., helminth.

Sex had a different impact depending on the immunoglobulin isotype. For antigen-specific IgG, there was a clear and consistent trend for higher antibody levels in females than males (Fig. 1 and Fig. S3A), which was statistically significant (*P* ≤ 0.05) for IgG to EXP1, AMA1, As37, AyCp2, NaAPR1, NaGST1, NaSAA2, and MEA in univariable models (Fig. 1). For total IgE, there was a trend in the opposite direction, with males exhibiting higher levels (Fig. 1 and Fig. S3A). When differences by sex were assessed stratifying by age group, there were no differences for antigen-specific IgG (data not shown), whereas total IgE was statistically significantly higher in male compared to female children (Fig. S3B).

There was a positive association between age and all antigen-specific IgG and total IgE levels (Fig. 1), with correlation coefficients ranging from rho = 0.31 to 0.64 for antigen-specific IgG and rho = 0.2 for total IgE (Fig. S4). IgG and IgE antibody levels rose until 15 to 24 years old and then remained relatively stable during the rest of adulthood (Fig. S5).

Being from a location with a high prevalence of malaria was associated with higher total IgE levels (*P* ≤ 0.001) (Fig. 1 and Fig. S6) and P. falciparum-specific IgG levels in univariable models, specifically for EXP1, AMA1, MSP1_42_, MSP2, and MSP5, but not with helminth-specific IgG levels (Fig. 1). Living in a location with a higher burden of helminth infection was associated with higher total IgE levels (*P* ≤ 0.05) (Fig. 1 and Fig. S7A), but not with helminth-specific nor P. falciparum-specific IgG levels (Fig. 1).

SES was negatively associated with IgG anti-EXP1, AMA1, MSP1 block 2, MSP1_42_, and NIE and total IgE (Fig. 1). Overall, there was a weak but significant negative correlation (rho = –0.17 to –0.1) between antigen-specific IgG levels and SES, whereas the negative correlation with total IgE levels was somewhat stronger (rho = –0.21) (Fig. S8).

Regarding water and sanitation metrics, individuals with a latrine at home had significantly lower total IgE levels than individuals without a latrine at home (*P* ≤ 0.001) (Fig. 1 and Fig. S7B), while specific-IgG levels were not significantly associated (Fig. 1). Additionally, living in a household with piped water inside and paying for water access were also significantly associated with lower total IgE levels compared to living in a household with piped water in the yard (*P* ≤ 0.01) or a household that did not pay for water (*P* ≤ 0.001) (Fig. 1, Fig. S7C and S7D). Piped water accessibility outside the household was associated with higher antigen-specific IgG levels against AMA1, EBA175, MSP1 block 2, MSP3, and MSP5 and no payment for water with IgG levels against α-gal, SSP2, LSA1, EXP1, MSP1 block 2, MSP1_42_, MSP3, Rh1, and NIE (*P* ≤ 0.05) (Fig. 1).

### Association of coexposure/coinfection with antibody levels.

**FIG 2:**
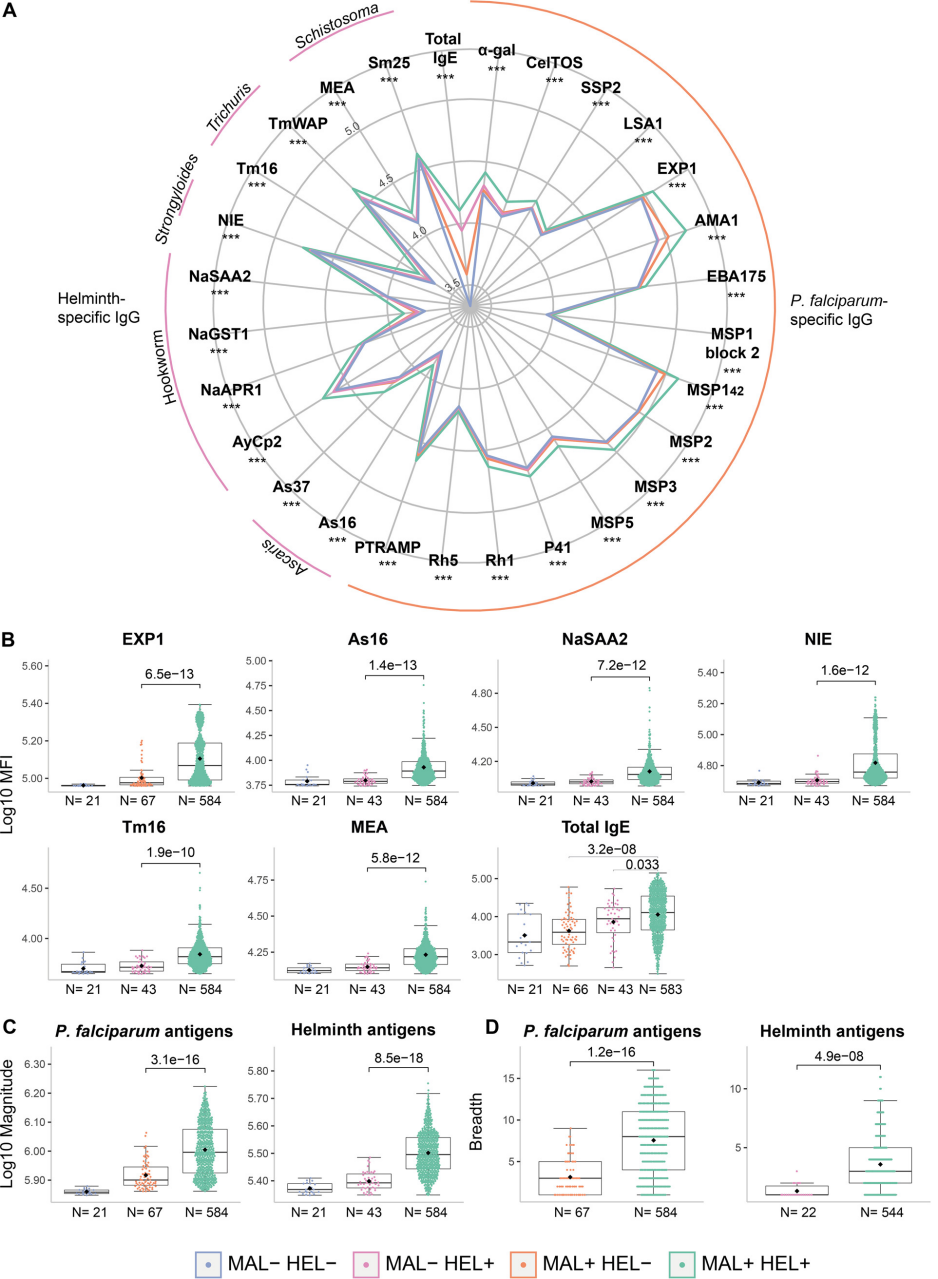
Comparison of antibody responses between exposure/infection groups. (A) Radarplot summarizing the antibody responses per exposure/infection group. In the vertices, the antibody responses are grouped by specific IgG against Plasmodium falciparum antigens, helminth antigens, or total IgE. The colored lines represent the median of antibody levels expressed as log_10_-transformed median fluorescence levels (MFI). For P. falciparum and helminth antigen-specific IgG, the medians of the MAL+ HEL+ group were compared with the median of the MAL+ HEL− or MAL− HEL+ groups, respectively, by Wilcoxon rank-sum test. For total IgE, the median of the MAL+ HEL+, MAL+ HEL−, and MAL− HEL+ groups were compared by Kruskall-Wallis test. Statistically significant differences are indicated with asterisks (***, *P*  ≤  0.001). The boxplots compare infection groups based on (B) the log_10_-transformed MFI specific IgG levels against representative antigens per parasite species and total IgE, (C) the log_10_-transformed magnitude of response (sum of MFI), and (D) the breadth of response (number of seropositive antigen-specific IgG responses). The boxplots represent the median (bold line), the mean (black diamond), the first and third quartiles (box), and the largest and smallest values within 1.5 times the interquartile range (whiskers). Data beyond the end of the whiskers are outliers. Statistical comparison between groups was performed by Wilcoxon rank-sum test and the adjusted *P values* by the Benjamini-Hochberg approach are shown. Study groups are shown in colors, no exposure/infection (violet) (MAL− HEL−); only exposure/infection with helminths (pink) (MAL− HEL+); only exposure/infection with P. falciparum (orange) (MAL+ HEL−) and coexposure/coinfection with P. falciparum and helminths (green) (MAL+ HEL+). The rest of the antigens are shown in Fig. S9.

The MAL+ HEL+ group consistently showed higher IgG levels against all P. falciparum and helminth antigens and higher total IgE levels (Fig. 2A and B, Fig. S9), magnitude (defined as the sum of antibody levels against P. falciparum or helminth antigens) (Fig. 2C) and breadth of response (defined as the number of P. falciparum or helminth seropositive responses) (Fig. 2D) compared with the MAL+ HEL− or MAL− HEL+ groups (*P* ≤ 0.001 except for IgE levels between MAL+ HEL+ and MAL-HEL+ where *P* = 0.033). Because all antibody levels increased with age, and there was a biased distribution of age groups across infection groups, this analysis was also performed stratified by age, where only the children had enough sample size in all the groups. The results in children confirmed the results in the whole cohort, with statistically significant differences in all comparisons except for IgG against highly immunogenic antigens such as AMA1, EBA175, and MSP1_42_, where after adjusting for multiple comparisons statistical significance disappeared, although a clear trend remained (Fig. S10).

**FIG 3:**
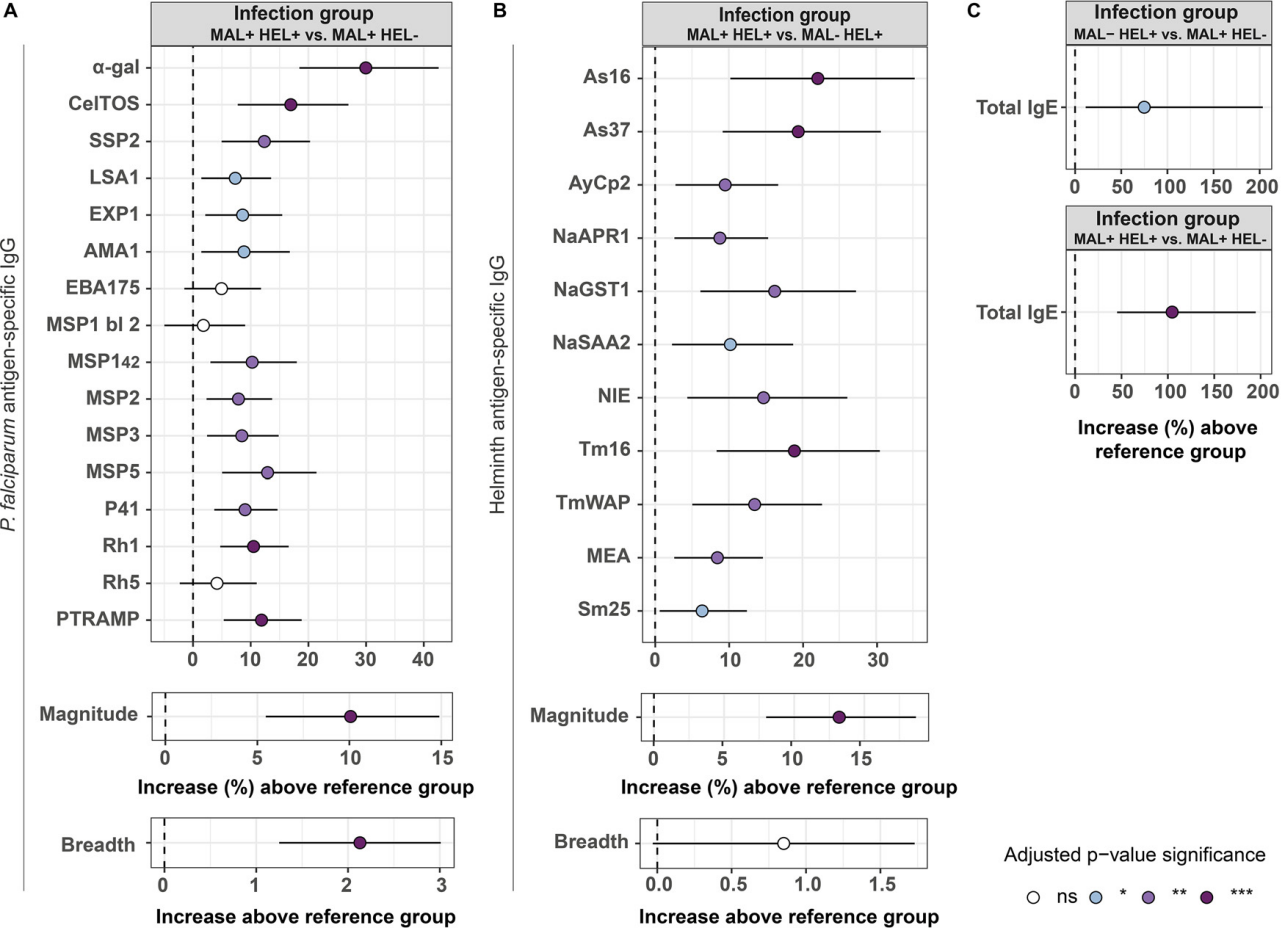
Association of exposure/infection group with antibody levels in multivariable linear regression models. Forest plots show the association of the MAL+ HEL+ group with (A) IgG levels to P. falciparum antigens, magnitude, and breadth of response or (B) IgG levels to helminth antigens, magnitude, and breadth of response. In (C), the association of MAL+ HEL+ group or the MAL− HEL+ group with IgE levels was assessed. The magnitude of response was calculated as the sum of all specific IgG levels to the different antigens belonging to P. falciparum or helminths. The breadth was defined as the number of seropositive antigen-specific IgG responses. Multivariable linear regression models were fitted to calculate the estimates (dots) and 95% confidence intervals (CI) (lines). The represented values are the transformed betas and CI showing the percentage increase in comparison with the reference category for IgG levels to antigens, total IgE and magnitude of response (log-linear models), and the untransformed betas and CI showing the increase in units in comparison with the reference category for the breadth of response (linear-linear models) (see “Statistical analysis” section for more details). The color of the dots represents the *P value* significance after adjustment for multiple testing by Benjamini-Hochberg, where ns denotes not significant and *, *P value* ≤ 0.05; **, *P value*  ≤  0.01; ***, *P value*  ≤  0.001. Models were adjusted by age and percentage of P. falciparum-infected individuals by location for P. falciparum antigens, age for helminth antigens, and sex, percentage of helminth-infected individuals by location, ownership of latrine, piped water accessibility, payment for water, and socioeconomic score for total IgE. MSP1 bl 2, MSP1 block 2; MAL+ HEL+, coexposure/coinfection with P. falciparum and helminths; MAL+ HEL−, only exposure/infection with P. falciparum*;* MAL− HEL+, only exposure/infection with helminths.

In multivariable analysis, linear regression models adjusted by their corresponding covariables revealed that the MAL+ HEL+ group had significantly higher antibody levels against most of the antigens, magnitude and breadth of response compared to single infection/exposure groups (MAL+ HEL− or MAL− HEL+) (Fig. 3).

For most P. falciparum antigens, IgG levels increased between 5% and 15% in the MAL+ HEL+ group compared with the MAL+ HEL− group, although it reached a 17% increase for CelTOS (95% CI, 7.750 to 26.932) and a 30% for α-gal (95% CI, 18.438 to 42.570) (Fig. 3A). The only antigens without a significant increase associated with coinfection were EBA175, MSP1 block 2, and Rh5 (Fig. 3A). The MAL+ HEL+ group resulted in an increase of up to a 10% (95% CI, 5.462 to 14.889) in the magnitude of response, and an increase of 2 in the total number of seropositive responses (95% CI, 1.254 to 3.001) (Fig. 3A).

Regarding helminth antigens, the increase in IgG levels in the MAL+ HEL+ group compared with the MAL− HEL+ group ranged from 6% for Sm25 (95% CI, 0.624 to 12.463) to 22% for As16 (95% CI, 10.213 to 35.249) (Fig. 3B). The magnitude increased up to a 13% (95% CI, 7.995 to18.684) while the breadth was not significantly affected (0.852; 95% CI, −0.030 to 1.734) (Fig. 3B).

For total IgE levels, MAL− HEL+ or MAL+ HEL+ groups were significantly associated with a 74% (95% CI, 6.193 to 185.409) or 106% (95% CI, 46.453 to 191.300) increase in total IgE levels, respectively, compared with the MAL+ HEL− group (Fig. 3C).

### Association of magnitude of response and total IgE with antibody levels.

We also performed additional multivariable models in the whole cohort using total IgE or the magnitude of response against malaria and helminth antigens as predictor exposure variables instead of exposure/infection groups (Fig. S11 and S12). Total IgE was significantly positively associated with the magnitude and breadth of response and IgG levels against P. falciparum and helminth antigens (Fig. S11). The magnitude of response against helminth antigens was also positively associated with IgG levels against P. falciparum antigens. The magnitude of response against P. falciparum antigens was also positively associated with IgG levels against helminth antigens, and both magnitudes with total IgE levels (Fig. S12). Lastly, as expected, the magnitude of response against helminth antigens was more associated with an increase in total IgE levels (31%; 95% CI, 22.314 to 39.504) than the magnitude of response against P. falciparum antigens (9%; 95% CI, 2.778 to 15.956) (Fig. S12).

### Association of coexposure/coinfection with susceptibility to infection.

**FIG 4:**
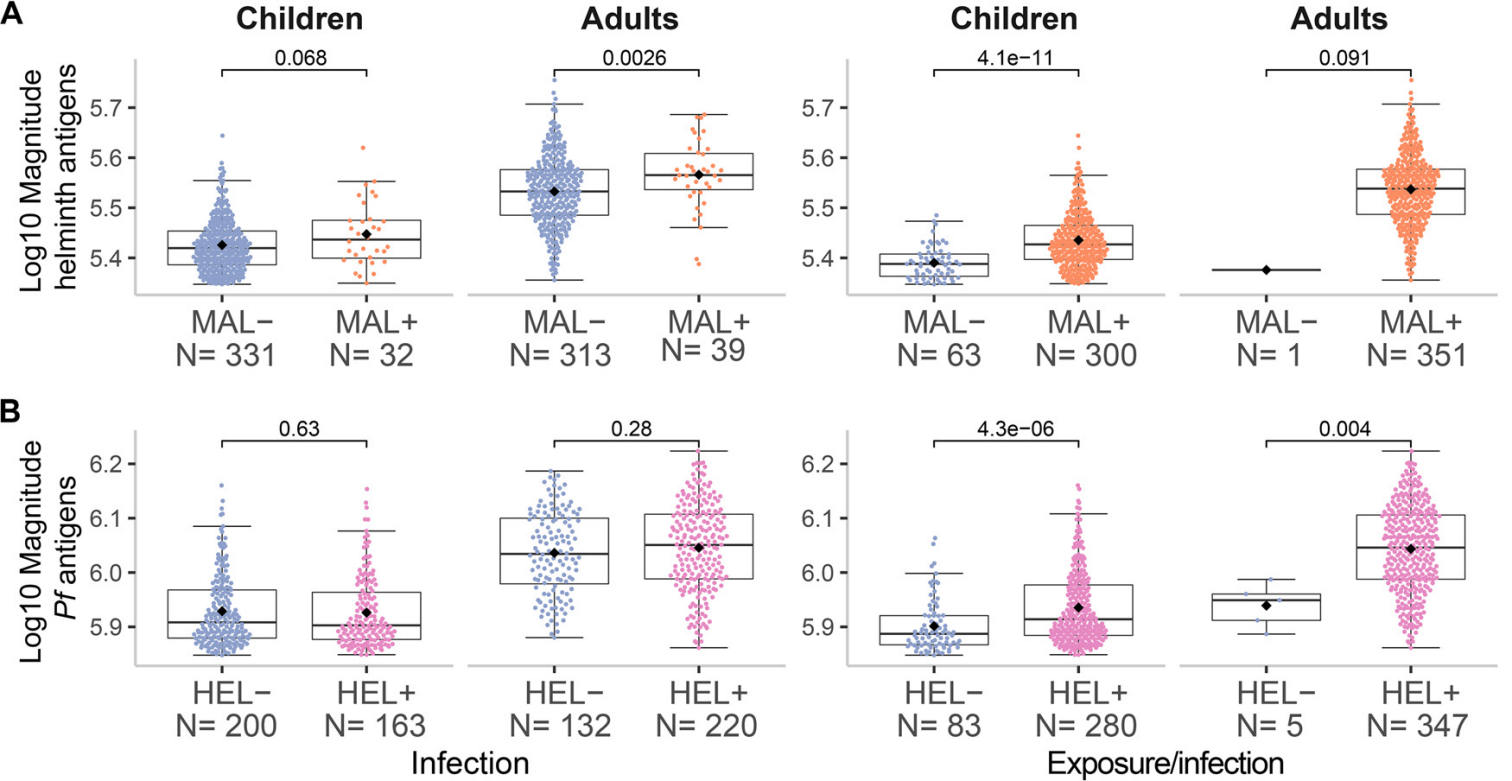
Magnitude of response by infection or exposure/infection with malaria or helminths. The magnitude of response represents the sum of all specific IgG responses against (A) helminth or (B) P. falciparum antigens. The *x*-axis shows the classification of samples into negative (−) or positive (+) based on either past exposure and/or present infection (detected by serology and molecular and/or microscopic diagnosis) or present infection (detected by molecular and/or microscopic diagnosis) for (A) malaria (MAL) or (B) helminths (HEL). The boxplots represent the median (bold line), the mean (black diamond), the first and third quartiles (box) and the largest and smallest values within 1.5 times the interquartile range (whiskers). Data beyond the end of the whiskers are outliers. Statistical comparison between groups was performed by Wilcoxon rank-sum test and the exact *P values* are shown.

Logistic regression analysis adjusted by their corresponding covariables showed a consistent trend for higher odds of having malaria or helminth infections if exposed to or currently having the other parasite type (Table S5). In support of this trend, a higher magnitude of antibody response to helminth or P. falciparum antigens was significantly associated with having P. falciparum or helminth infection, respectively (Fig. 4). In addition, individuals exposed/infected with a greater number of helminth species had more odds to be infected with P. falciparum (OR = 1.607; 95% CI, 1.214 to 2.167) (*P* ≤ 0.001), and individuals exposed/infected with P. falciparum were infected with more helminth species (β = 0.637; 95% CI, 0.316 to 0.958) (*P* ≤ 0.001). Furthermore, there was a statistically significant higher proportion of individuals exposed/infected with multiple helminth species in the MAL+ HEL+ group than in the MAL− HEL+ group (*P* ≤ 0.001) (Table S2).

### Association of coexposure/coinfection with parasite burden.

**FIG 5:**
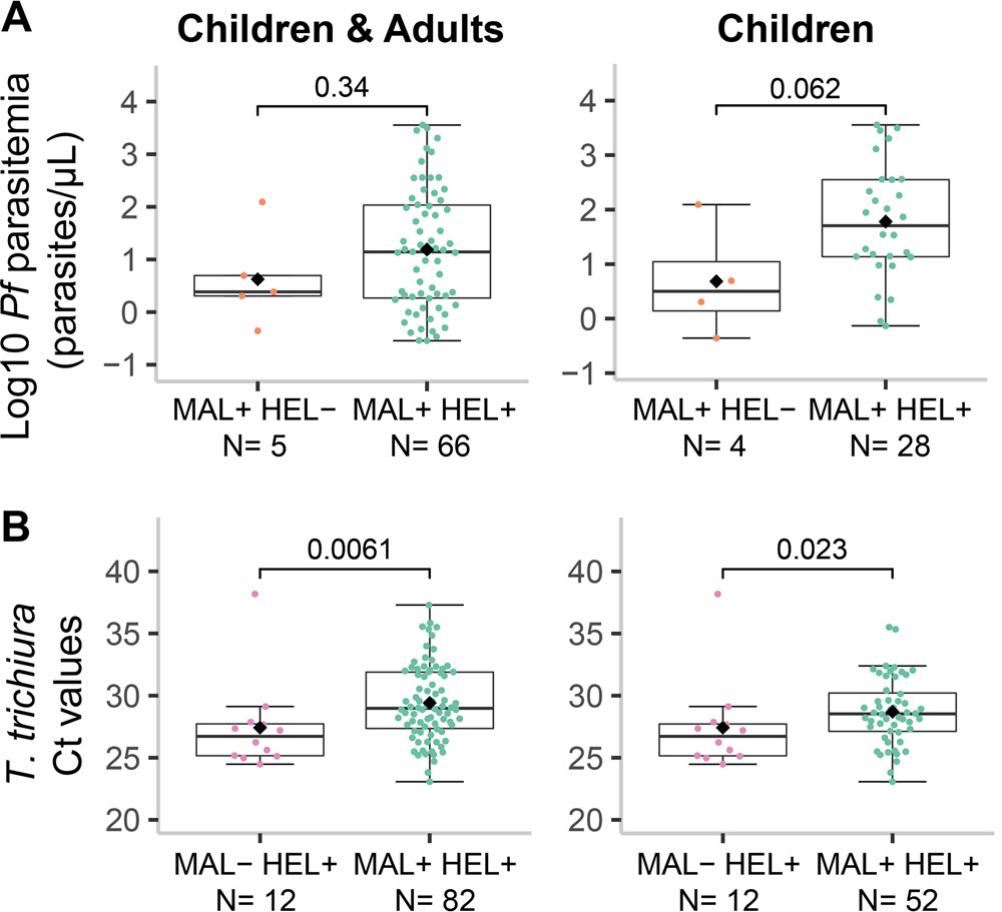
Parasite burden by exposure/infection groups. The levels of parasite burden are represented as (A) the log_10_-transformed parasitemia of Plasmodium falciparum (parasites/μL) or (B) the Trichuris trichiura Ct values, and are compared between the coinfection group (MAL+ HEL+) and the only malaria group (MAL+ HEL−) for P. falciparum, or the only helminths group (MAL− HEL+) for T. trichiura. For each parasite, data for all individuals with parasite burden information or only children are displayed. The boxplots represent the median (bold line), the mean (black diamond), the first and third quartiles (box) and the largest and smallest values within 1.5 times the interquartile range (whiskers). Data beyond the end of the whiskers are outliers. Statistical comparison between groups was performed by Wilcoxon rank-sum test and the exact *P values* are shown. See Fig. S13 for the plots of the rest of the parasites.

Next, we evaluated whether being exposed and/or having a coinfection had an impact on parasite burden. In the case of malaria, 93% of the people with current P. falciparum infection were coexposed/coinfected with helminths (66/71), leaving the single infection group with a very reduced sample size. In this case, the coexposure/coinfection group was not significantly associated (*P* = 0.34) with P. falciparum parasitemia (parasites/μL) (Fig. 5A). However, because immunity develops over time and most of the coexposed/coinfected individuals were adults, we stratified by age group, which resulted in a trend (*P* = 0.062) for higher parasitemia in the coexposure/coinfection group compared to the malaria only group in children (Fig. 5A). In the case of helminths, coexposure/coinfection had a significant protective effect on parasite burden (expressed as qPCR Ct values) of T. trichiura (*P* ≤ 0.01), also after stratification by age group (*P* ≤ 0.05) (Fig. 5B). For hookworm and A. lumbricoides there was no significant association between coexposure/coinfection and parasite burden (Fig. S13), while for Schistosoma spp. and S. stercoralis comparisons were not possible since all individuals infected with these species were in the coexposure/coinfection group.

### Association of antibody levels with parasite burden.

To investigate the associations of antibody responses with parasite burden and their possible involvement in protection, we performed linear regression models adjusted by age. For P. falciparum parasitemia, there was a significant (raw *P* ≤ 0.05) positive association of specific IgG responses to CelTOS (17.793; 95% CI, 2.99 to 35.634), SSP2 (22.229; 95% CI, 1.38 to 47.361), AMA1 (34.277; 95% CI , 2.410 to 76.060), MSP5 (26.824; 95% CI , 5.440 to 52.545) and the breadth of response to P. falciparum antigens (20.838, 95% CI, 1.111 to 44.415) only before adjustment for multiple comparisons.

As for helminths, we found significant negative associations of qPCR Ct values with total IgE (−0.085; 95% CI, −0.141 to −0.028) for hookworm, specific IgG to Sm25 (−0.963; 95% CI, −1.573 to −0.353) and MEA (–1.065; 95% CI, −1.862 to −0.268) for *Schistosoma* spp. and NIE for S. stercoralis (−0.542; 95% CI, −0.828 to −0.256). These negative associations with Ct values are indicative of a positive association with parasite burden. We found no association between A. lumbricoides and T. trichiura antibody levels and Ct values.

## DISCUSSION

In this study we found that 83% of a cohort of children and adults from Southern Mozambique was infected or had been exposed to P. falciparum and helminths, mainly hookworm. Our main finding was that coexposure/coinfection was associated with increased antibody levels, magnitude, and breadth of response to both types of parasites compared with the single exposure/infection groups.

We initially expected a deviation in the coexposure/coinfection group toward one of the branches of the immune response, which would result in reduced antibody production of the specificity and type required for one or other type of infection. On the contrary, we found that antigen-specific IgG and total IgE responses were higher in the coexposure/coinfection group compared to the single exposure/infection groups. For P. falciparum, this is consistent with previous studies that reported increased P. falciparum-specific IgG1, IgG2, and IgG3 responses in coinfection with *Schistosoma haematobium* (40, 41). In contrast, other studies have reported a detrimental effect of coinfection with helminths in the humoral response to malaria with a reduction in P. falciparum-specific IgG (42), IgG1 and IgG3 (43, 44), and an increase in the non-cytophilic IgG4 (43). As for helminths, the effect of P. falciparum infection on antibody levels has been mainly restricted to Schistosoma spp. and, in line with our results, coinfection with P. falciparum has been associated with an increase in both antigen-specific IgG and IgE responses to Schistosoma spp. antigens compared with the single infection group (45). Does this increase mean there is a synergy at the immune humoral compartment or does it simply reflect an increased susceptibility, and therefore exposure to infections in the coinfection group?

Three main concepts support the synergy theory. First, the fact that both types of parasites are able to induce Th2 responses, at least at some point during the course of infection, could favor higher levels of antibodies since antibody production requires the aid of classical Th2-like cytokines (46, 47). However, whether these antibodies are protective or not needs to be evaluated in longitudinal studies. In the case of P. falciparum, the synergy hypothesis is supported by an increase in P. falciparum-specific IgG1 and IgG3 during coinfection with *S. haematobium* (40). Similarly, P. falciparum coinfection with hookworm increased IgG1 and IgG3 levels to the GMZ2 vaccine and related antigens, while deworming treatment reduced these IgG1 and IgG3 levels (48). Second, our observation of higher magnitude and breadth of response to P. falciparum and helminth antigens in coexposed/coinfected individuals could be explained by the ability of P. falciparum and helminths to induce polyclonal B cell responses (49, 50), which is particularly evident for IgE during helminth infections (10). This phenomenon could be a strategy to avoid host-specific immune responses by “diluting” antibodies against important epitopes for protection (51) and bystander antigens in coinfections could be affected by this spillover effect (10). Finally, feedback enhancement has been proposed as a mechanism by which IgE amplifies IgG production (52). Specific IgE would bind to CD23 receptors on B cells, increasing the internalization of antibody-antigen complexes and the presentation to T cells, ultimately favoring IgG production.

Nonetheless, our results point toward another more plausible option, which is that antibody responses are a consequence of increased exposure due to higher susceptibility to infection in the coexposure/coinfection group. Firstly, antibody levels were positively associated with parasite burden, indicating that they reflect exposure rather than protection. We checked whether differences in the proportion of current infections between groups could be the cause due to boosting of the immune response, but there were no differences between groups except for T. trichiura, whose proportion was higher in the helminth single exposure/infection group. Secondly, our results suggest that individuals exposed to or infected with P. falciparum or helminths were more likely to have been exposed to or be infected with the other parasite. In addition, exposure/infection with an increasing number of helminth species was associated with being exposed to or infected with P. falciparum and *vice versa.* In line with this, others were able to confirm that infection with helminths in general, or more helminth species, increases P. falciparum density or the odds of P. falciparum infection (15, 18–20, 34, 53). This observation is consistent with the concept of hypersusceptible hosts, who are individuals infected to a greater degree as a result of exposure to pathogens or underlying conditions.

Therefore, if higher antibody levels simply reflect higher exposure, is there any underlying factor that renders these individuals in the coexposure/coinfection group more prone to multiple infections? It has been suggested that factors favoring coinfections may be common socioeconomic or environmental factors rather than true immunological interactions (54, 55). One of the strengths of this study is that we accounted for socioeconomical and water and sanitation factors, besides demographic variables, that could influence the exposure to the parasites. Among all variables, age had the strongest association with antibody levels. We clearly showed that the acquisition of immunity is similar across all antibody responses, with levels rising during childhood and stabilizing during adulthood. This is consistent with a cumulative exposure over time, which resulted in a clear age bias whereby most of the adults were in the coexposure/coinfection group. Socioeconomic and water and sanitation metrics had the strongest associations with total IgE, with higher SES and improved water and sanitation conditions showing the lowest IgE levels. This is consistent with higher exposure to helminths, potent inducers of IgE, under poor sanitation and hygiene conditions, considering that transmission of STH and *Schistosoma* spp. relies on the fecal spread of eggs and larvae. It is noteworthy that also specific IgG levels against some P. falciparum and helminth antigens were affected by these variables, suggesting shared transmission risks, such as fecally contaminated water in the household surroundings that can serve as a reservoir of helminth eggs and mosquito larvae. However, only age was significantly higher in the coexposure/coinfection group compared with the rest. Therefore, immunological permissiveness seems to be a likely cause for the observed increase in antibody responses. Nonetheless, we cannot rule out other underlying conditions, such as malnutrition, that could make this group of individuals more susceptible to infections.

It is remarkable that in the absence of infection and exposure to helminths, P. falciparum seems to be an inducer of total IgE. Additionally, in the coexposure/coinfection group, total IgE levels were also higher than in the helminth single exposure/infection group. This suggests that P. falciparum also contributes to these IgE levels, because exposure to or coinfection with P. falciparum does not seem to increase helminth burden. Interestingly, another study has also found elevated total IgE levels in malaria patients regardless of helminth coinfection (56). IgE is a neglected isotype in the field of malaria, with limited studies focusing on it. As a result, the role IgE plays in protection or susceptibility remains to be elucidated. Some have argued it has a protective role (57–60) while others indicate that it is involved in malaria pathogenesis (61–67). In our linear models, IgE was positively associated with P. falciparum-specific IgG levels that have been shown to reflect exposure rather than protection. This indirectly supports the idea of a non-protective role of IgE in terms of P. falciparum infection. However, we could not assess its implication in clinical outcomes or associations for P. falciparum antigen-specific IgE, which might be different than those of total IgE (60) probably due to the polyclonal, nonspecific nature of total IgE responses (68).

A caveat of this study is the inability to determine causality due to its cross-sectional nature. A longitudinal design, on the other hand, would be more appropriate to determine whether people with P. falciparum or helminths are more susceptible to subsequent infections. Furthermore, this study only included asymptomatic malaria cases, which may be hindering the detection of important associations with disease severity and masking the true burden of coinfections. Nonetheless, even with only asymptomatic cases, we were able to identify significant associations that suggest a higher susceptibility to infection. Therefore, we expect that the inclusion of symptomatic cases would only accentuate these associations. An additional limitation is the pooling of all helminth species together due to the small sample size of the groups at the species level, possibly resulting in masked effects of individual species (69). Lastly, we could not assess if there was certain cross-reactivity between antibodies against helminth and P. falciparum antigens, which is unlikely but could have affected the increase of antibody levels observed in the coexposure/coinfection group.

In conclusion, this study shows a significant increase in antibody responses in individuals coexposed to or coinfected with P. falciparum and helminths in comparison with individuals exposed and/or infected with only one of these parasites. Although we cannot rule out a possible synergistic effect of these parasites at the immunological level or a certain degree of cross-reactivity, our results suggest that the increase in the antibody responses is due to a more permissive immune environment to infection in the host. Additional studies are needed for a better understanding of the mechanisms involved during coinfections at the immunological level, but also that lead to coinfections in the first place. We also highlight the importance of taking previous exposure into account, especially in endemic areas where continuous infections imprint and shape the immune system. For future studies, we suggest including the measurement of antigen-specific IgE and IgG subclasses, cytokines, and cell populations. Deciphering the implications of coinfections deserves attention because accounting for the real interactions that occur in nature could improve the design of integrated disease control strategies.

## MATERIALS AND METHODS

### Study design.

This immunological analysis was done in the context of a community-based, case-cohort study (MARS) that recruited 819 individuals. MARS aimed to design and implement a surveillance platform to identify and characterize genotypically and phenotypically anthelmintic resistance in Manhiça district, Maputo province, Southern Mozambique (70). Households (*N*  =  384) from Manhiça district were randomly selected using a grid-sampling methodology. A field worker visited the household and invited two people to participate, one between the ages of 5 and 15 and the other over 15 years old. Therefore, MARS included inhabitants aged 5 years or above, censed in the Manhiça Health Research Centre (CISM) Demographic Surveillance System, who had not taken anthelmintics at any time during the previous 30 days. The locations included were 3 Fevereiro, Calanga, Ilha Josina, Maluana, Manhiça-sede, and Xinavane. The study protocol was approved by the National Bioethics Committee for Health in Mozambique with the approval code number Ref.:517/CNBS/17. Informed consent from individuals who were willing to participate was obtained and, in the case of children, from their parents or guardians. Additionally, participants aged 15 to 17 also gave informed assent.

The final sample size for this analysis was 715. One-hundred and three samples were excluded from the analysis because they were either not shipped to Barcelona (Spain) (*N* = 5) or due to study withdrawal (*N* = 25), sample insufficiency (*N* = 4) or incorrect serum elution (*N* = 69).

### Detection and quantification of P. falciparum and helminth infections.

Stool, urine, and blood samples were collected from participants for laboratory analyses. Microscopic diagnosis of N. americanus, *A. duodenale*, *T. trichiura*, *A. lumbricoides*, and S. mansoni was performed at the CISM by search of eggs in two stool samples using duplicate Kato-Katz thick smear technique. S. stercoralis was detected in two stool samples using the Telemann technique. *S. haematobium* infection was detected by urine filtration. In addition, molecular diagnosis of infections caused by the aforementioned pathogens was performed at the Leiden University Medical Center (the Netherlands). In brief, the presence of parasite-specific DNA was determined in one stool sample per participant by multiplex semiquantitative real-time Polymerase chain reactions (qPCR), using sequences of primers and probes and a general PCR set-up as described before, in which two different multiplex detection panels were used; panel I targeted *Schistosoma* spp. and *T. trichiura*, and panel II targeted *A. duodenale*, N. americanus, *A. lumbricoides*, and S. stercoralis (71). Individuals were considered to be positive for helminth infection if they had a positive result in at least one of the techniques (microscopy in urine or in at least one of the stool samples, or qPCR).

Capillary blood samples were collected through finger pricking on Whatman 903 filter paper to obtain dried blood spots (DBS). P. falciparum infection was determined at the Barcelona Institute for Global Health (ISGlobal) by 18S rRNA gene detection through qPCR from DBS samples. Briefly, six DBS punches of 3 mm in diameter were used for DNA extraction by the Chelex method (72) along with negative controls (NC) consisting of DBS from noninfected erythrocytes (Banc de Sang i Teixits [BST], Barcelona, Spain) and positive controls (PC) of DBS with P. falciparum (3D7 isolate) ring-infected red blood cells diluted in whole blood. Purified DNA templates were amplified following a previously described method (73). A NC with ddH2O instead of DNA template as well as the punch controls were included in each plate. Each specimen was run in duplicate and the standard curve in triplicate for each plate. Parasite density was quantified by Ct interpolation with a standard curve ranging from 18,000 to 1.8 parasites/μL.

### Measurement of antibody responses.

**(i) Antigen panel.** A total of 27 antigens, 16 from P. falciparum (α-gal, CelTOS, SSP2, LSA1, EXP1, AMA1 FVO, EBA175 reg2 PfF2, MSP1 block 2 MAD20, MSP1_42_ 3D7, MSP2 full-length CH150, MSP3 3D7, MSP5, P41, Rh1, Rh5, and PTRAMP) and 11 from helminths (STH [NaGST1, NaAPR1, NaSAA2, AyCp2, TmWAP, Tm16, As16, As37, NIE] and *Schistosoma* spp. [MEA, Sm25]), were selected based on their important role as vaccine candidates and markers of exposure (Table S6). In addition, some of them such as NIE and most P. falciparum antigens have a moderate performance as markers of current infection (0.7> AUC ≤ 0.9) (data not shown). The antigens in the panel are representative of the different stages of P. falciparum life cycle and of the most common intestinal helminth infections. In addition, glutathione *S*-transferase (GST) was added to the panel as a control for unspecific binding to this tag fused to MSP1 and MSP2 antigens.

As16, As37, AyCp2, NaAPR1, NaGST1, NaSAA2, Tm16, and TmWAP were expressed and purified from yeast Pichia pastoris X-33 culture at Baylor University (USA). NIE was expressed in E. coli by Sukwan Handali (Centers for Disease Control and Prevention, USA) from a plasmid clone kindly provided by Thomas Nutman (National Institute of Health and National Institute of Allergy and Infectious Diseases, USA); MEA and Sm25 were produced in yeast and E. coli, respectively, and purchased from MyBioSource (USA); α-gal from Dextra Laboratories (UK); and CelTOS, SSP2, LSA1, and EXP1 from Sanaria (USA) expressed in Pichia pastoris. AMA1 FVO, MSP1_42_ 3D7, and GST were expressed in E. coli at Walter Reed Army Institute of Research (USA); MSP5 expressed in E. coli at Monash University (Australia); MSP1 block 2 MAD20 and MSP2 full-length CH150 expressed in E. coli at University of Edinburgh (UK) and EBA175 R2 F2, Rh1, Rh5, PTRAMP, and P41 expressed in E. coli at International Centre for Genetic Engineering and Biotechnology (India).

### (ii) Serum elution from DBS.

Antibodies were obtained from DBS samples through an adaptation of a described method (74). Briefly, serum was eluted from 4 DBS punches, collected as described before, and incubated with 100 μL of PBS-BN (filtrated PBS with 1% BSA and 0.05% sodium azide; ref. S8032, MilliporeSigma, St. Louis, USA) and 0.05% Tween20 (ref. P1379, MilliporeSigma, St. Louis, USA) at 4°C overnight (ON) with gentle mixing (∼600 rpm). The next day, the tubes were centrifuged at 10,000 rpm for 10 min and the supernatant containing the eluted serum was transferred into a new tube, ready for serological measurement. Assuming a hematocrit of 50%, the eluted blood proteins concentration was equivalent to a 1:25 plasma dilution. Samples with reddish-brown spots against a pale background were discarded due to incorrect elution. To assure the validity of the punch and the serum elution procedure, a NC of filter paper without DBS and three elution control (EC) samples with known antibody levels (volunteer donors) were included in each elution batch.

### (iii) Luminex.

Quantitative suspension array technology (qSAT) assays were used to measure P. falciparum and helminth specific-IgG and total IgE as described previously (75). Briefly, the antigens and a capture anti-human IgE (Mouse anti-Human IgE Fc) (ref. ab99834, Abcam PLC, Cambridge, UK) were coupled to magnetic MagPlex 6.5 μm COOH-microspheres (Luminex Corporation, Austin, USA) at the optimal coupling conditions (Table S7). Coupled beads were validated (in multiplex in the case of antigen-specific IgG) by measuring antigen-specific IgG and total IgE in serial dilutions of a PC. The antigen-coupled beads were used in multiplex at 1,000 beads per region per sample, while the anti-human IgE-coupled beads were used in singleplex at 3,000 beads per sample.

Coupled beads were incubated with eluted serum samples, eluted controls, a PC curve, and blanks in 96-well μClear flat-bottom plates (ref. 655096, Greiner Bio-One, Frickenhausen, Germany) at 4°C ON in a shaker at 600 rpm. For the antigen-specific IgG quantification, we assayed the samples at 1:100 and 1:2,000 dilutions, and included a WHO malaria pool was used as PC [First WHO Reference Reagent for Anti-malaria (P. falciparum) human serum, NIBSC code: 10/198 (NIBSC, Herts, UK)] at 12 serial dilutions (3-fold) starting at 1:100. For total IgE quantification, we assayed the samples at 1:50 dilution and, included a WHO IgE PC (Third WHO International Standard Immunoglobulin E, human serum, NIBSC code: 11/234; NIBSC, Herts, UK) at 12 serial dilutions (2-fold) starting at 1/50. After the incubation of coupled beads with samples, beads were washed three times with PBS + 0.05% Tween20, using a manual magnetic washer platform (ref. 40–285, Bio-Rad, Hercules, USA), and the biotinylated secondary anti-human IgG (ref. B1140-1ML, MilliporeSigma, St. Louis, MO, USA) and the anti-human IgE (ref. A18803, Invitrogen, Waltham, USA) antibodies were added at 1:1,250 and 1:100 dilutions, respectively, and incubated for 45 min at room temperature (RT) shaking at 600 rpm. Then, beads were washed three times and streptavidin-R-phycoerythrin (ref. 42250-1ML, MilliporeSigma, St. Louis, USA) was added at 1:1,000 dilution and incubated at RT for 30 min at 600 rpm. Finally, beads were washed, resuspended, and at least 50 beads per analyte and sample were acquired in a FlexMap 3D xMAP instrument (Luminex Corporation, Austin, Texas, USA). Crude median fluorescent intensities (MFI) and background fluorescence from blank wells were exported using the xPONENT software v.4.3 (Luminex Corporation, Austin, Texas, USA).

### Determination of seropositivity.

Gaussian mixture models were used to estimate a seropositivity threshold for each antigen-specific IgG and total IgE response and classify the samples into positive and negative by serology. The models were estimated by the expectation-maximization (EM) algorithm. For each antigen, two models were fitted assuming either equal or unequal variances, and each of those two models was built with two clusters for classification. The two clusters of classification are meant to represent the distribution of seropositive and seronegative samples. For each antigen, classification of samples was done using the model showing the optimal Bayesian information criterion (BIC). Briefly, the EM algorithm is an unsupervised clustering algorithm that works in two steps. The first step is the expectation step, where the data are assigned to the closest centroid of the two clusters. The second step is the maximization step, where the centroids of the clusters are recalculated with the newly assigned data. These two steps are repeated until the model converges. The models were fitted using the R CRAN package mclust (76).

### Definition of study groups.

Study participants were classified into study groups based on diagnosis of current infection (defined by microscopy and/or qPCR) and prior exposure (defined by seropositivity to any of the antigens for each type of parasite). The resulting groups were: (i) no exposure/infection (MAL− HEL−), which included people that have not been exposed to or were not currently infected with P. falciparum nor helminths; (ii) single exposure/infections (MAL− HEL+ or MAL+ HEL−), which included people that have been exposed to and/or currently infected with only one type of parasite; and (iii) coexposure/coinfection (MAL+ HEL+), which included people that have been exposed to and/or currently infected with both types of parasites.

### Definition of study variables.

In this study, we included demographic variables such as sex, age, age group (children, 5 to 15 years old or adults, ≥15 years old) and percentage of infection by location. The latter was calculated based on the P. falciparum or helminth percentage of infection in each location of the study. Then, participants were classified into P. falciparum or helminth “high” and “low” groups of locations defined according to a higher or lower percentage of P. falciparum or helminths compared with the overall median by type of parasite (Table S4). Additionally, we also included socioeconomic (socioeconomic score), water (accessibility to piped water, payment for water), and sanitation (ownership of latrine) variables (70). Finally, we calculated the magnitude and breadth of response to P. falciparum and helminth antigens as previously described (77). The magnitude of response corresponds to the sum of all specific IgG levels (MFI) to the different antigens belonging to P. falciparum or helminths, and the breadth of response is defined as the number of antigens with seropositive responses.

### Statistical analysis.

To determine the seropositivity and perform the statistical analysis, normalized and dilution corrected data (see supplementary material) were log_10_-transformed. The Shapiro-Wilk test was used to check the normality of the data, and because most of the data did not follow a normal distribution, nonparametric tests were used. Then, we explored differences in the distribution of study variables across study groups. For continuous variables, the median and first and third quartiles were calculated and differences in the median between more than two groups were assessed by Kruskall-Wallis test followed by pairwise comparisons by Wilcoxon rank-sum test and Benjamini-Hochberg (BH) adjustment for multiple testing. For categorical variables, differences in proportions between groups were calculated by chi-square test or Fisher's exact test when applicable. This was automatically determined by the R CRAN package compareGroups (78). For comparisons between two groups, we applied the Wilcoxon rank-sum test and, in the case of antigen-specific IgG responses, *P values* were adjusted for multiple testing by the BH. Correlations between variables were assessed with the Spearman’s rank correlation coefficient ρ (rho). For Spearman’s test, *P values* were computed via the asymptotic *t* approximation.

For regression models, assumptions on the data were checked by exploratory plots. For linear regression models, we checked the linearity of the data, normality of residuals, homogeneity of residuals variance, and independence of residuals error terms. For logistic regression models, we checked the linearity between the logit of the outcome and each predictor variable, the presence of influential values and multicollinearity among the predictor variables. All the assumptions were met without the presence of extreme patterns, except for normality of the residuals in most cases.

The first linear regression models were performed to assess the association between antibody responses (including antigen-specific IgG levels, total IgE levels, magnitude of response, and breadth of response) as outcome variables and sex, log_10_ age, percentage of infected individuals by location, SES, accessibility to piped water, payment for water, and ownership of latrine in univariable models. The second linear regression models were multivariable models fitted to assess the association between antibody responses as outcome variables and three predictor variables: (i) the exposure/infection status as categorical variable (coexposed/coinfected or single exposed/infected); (ii) log_10_ total IgE levels as continuous variable; or (iii) the magnitude of response to P. falciparum or helminth antigens as continuous variable. We evaluated the association of these three predictor variables independently by building three different base models. To select the adjusting covariables, we first selected those variables significantly associated with each block of antigens by univariable models. Then, we explored all possible combinations of the predictor covariables with the base models and chose per block of antigens those combinations that provided the simplest models (based on BIC) without a significant compromise of the Akaike information criterion (AIC) and adjusted r-square. The third linear regression models explored the association between P. falciparum exposure/infection (predictor variable) and the number of helminth species (outcome variable) adjusted by the corresponding covariables selected as explained above. The fourth linear regression models were performed to assess the association between parasite burden as outcome variable and antibody responses as predictor variables adjusted by age. We adjusted for multiple comparisons by BH, where the different antibody responses were penalized.

The logistic regression models evaluated the association of exposure/infection with either P. falciparum or helminths (predictor variable) with being exposed/infected with the counterpart parasite (outcome variable). For P. falciparum, we also evaluated by logistic regression the association of the number of helminth species (predictor variable) with exposure/infection with P. falciparum (outcome variable). The selection of adjusting covariables was done based on significant associations by univariable models.

The resulting betas and 95% confidence intervals (CI) obtained in each linear regression model were transformed when appropriate for interpretation if the outcome (log-linear model), predictor (linear-log model), or both (log-log model) variables were log_10_-transformed. For log-log models, the transformed values (%) were calculated with the formula {[10^(beta*log_10_1.1)]−1}*100. This represents the effect (in percentage) on the outcome variable of a 10% increase in the corresponding predictor variable. For log-linear models, the transformed values (%) were calculated with the formula [(10^beta)-1]*100. This represents the effect (in percentage) on the outcome variable of an increase in one unit of the predictor variable, or with respect to the reference category in cases of categorical variables. Finally, for linear-log models, the transformed value was calculated with the formula beta*log_10_1.1. This represents the additive effect on the outcome variable of a 10% increase in the corresponding predictor variable.

Adjusted and raw (if not adjusted for multiple testing) *P values* of ≤ 0.05 were considered statistically significant. All data processing and statistical analyses were performed using the statistical software R version 4.0.3 and R Studio Version 1.1.463. Other R CRAN packages not mentioned above but used to manage data, generate tables, and plots were tidyverse (79), ggiraphExtra (80), ggpubr (81), and ggbeeswarm (82).
