# Supplementary material for: Plasmodium falciparum and Helminth Coinfections Increase IgE and Parasite-Specific IgG Responses
Source: Microbiol Spectr. 2021 Dec 8;9(3):e01109-21. doi: 10.1128/Spectrum.01109-21 (PMC8653825; doi:10.1128/Spectrum.01109-21)
Supplement: SUPPLEMENTAL FILE 1 — Supplemental material. Download SPECTRUM01109-21_Supp_1_seq9.pdf, PDF file, 2.9 MB [file spectrum01109-21_supp_1_seq9.pdf]

SUPPLEMENTARY TABLES

Table S1. Percentage of exposure, infection or both by parasite species and groups.

	**Total**	**MAL- HEL- (N=21)**	**MAL- HEL+ (N=43)**	**MAL+ HEL- (N=67)**	**MAL+ HEL+ (N=584)**	**p-value**
**Present infection**						
Hookworm	201 (28.11%)	0 (0.00%)	17 (39.5%)	0 (0.00%)	184 (31.5%)	0.358
T. trichiura	103 (14.41%)	0 (0.00%)	14 (32.6%)	0 (0.00%)	89 (15.2%)	**0.008**
A. lumbricoides	53 (7.41%)	0 (0.00%)	5 (11.6%)	0 (0.00%)	48 (8.22%)	0.399
S. stercoralis	74 (10.35%)	0 (0.00%)	1 (2.33%)	0 (0.00%)	73 (12.5%)	0.158
Schistosoma spp.	86 (12.03%)	0 (0.00%)	1 (2.33%)	0 (0.00%)	85 (14.6%)	0.097
Any helminth	383 (53.57%)	0 (0.00%)	31 (72.1%)	0 (0.00%)	352 (60.3%)	0.170
P. falciparum	71 (9.93%)	0 (0.00%)	0 (0.00%)	5 (7.46%)	66 (11.3%)	0.455
**Positive serology**						
Hookworm	497 (69.51%)	0 (0.00%)	17 (39.5%)	0 (0.00%)	480 (82.2%)	**≤0.001**
T. trichiura	377 (52.73%)	0 (0.00%)	7 (16.3%)	0 (0.00%)	370 (63.4%)	**≤0.001**
A. lumbricoides	148 (20.7%)	0 (0.00%)	0 (0.00%)	0 (0.00%)	148 (25.3%)	**≤0.001**
S. stercoralis	195 (27.27%)	0 (0.00%)	1 (2.33%)	0 (0.00%)	194 (33.2%)	**≤0.001**
Schistosoma spp.	314 (43.92%)	0 (0.00%)	4 (9.30%)	0 (0.00%)	310 (53.1%)	**≤0.001**
Any helminth	566 (79.16%)	0 (0.00%)	22 (51.2%)	0 (0.00%)	544 (93.2%)	**≤0.001**
P. falciparum	651 (91.05%)	0 (0.00%)	0 (0.00%)	67(100%)	584 (100%)	NA
**Exposure/infection**						
Hookworm	540 (75.52%)	0 (0.00%)	31 (72.1%)	0 (0.00%)	509 (87.2%)	**0.011**
T. trichiura	426 (59.58%)	0 (0.00%)	20 (46.5%)	0 (0.00%)	406 (69.5%)	**0.003**
A. lumbricoides	190 (26.57%)	0 (0.00%)	5 (11.6%)	0 (0.00%)	185 (31.7%)	**0.012**
S. stercoralis	210 (29.37%)	0 (0.00%)	2 (4.65%)	0 (0.00%)	208 (35.6%)	**≤0.001**
Schistosoma spp.	339 (47.41%)	0 (0.00%)	5 (11.6%)	0 (0.00%)	334 (57.2%)	**≤0.001**
Any helminth	627 (87.69%)	0 (0.00%)	43 (100%)	0 (0.00%)	584 (100%)	NA
P. falciparum	651 (91.05%)	0 (0.00%)	0 (0.00%)	67(100%)	584 (100%)	NA

Table S2. Percentage of single or multiple helminth species exposure, infection or both by helminth positive groups

Helminth species	Total	MAL- HEL+	MAL+ HEL+	p-value
**Present infection**				
**Single**	271 (70.76%)	25 (80.6%)	246 (69.9%)	0.291
**Multiple**	112 (29.24%)	6 (19.4%)	106 (30.1%)	
**Total**	383 (100%)	31 (100%)	352 (100%)	
**Positive serology**				
**Single**	129 (22.79%)	16 (72.7%)	113 (20.8%)	**≤0.001**
**Multiple**	437 (77.21%)	6 (27.3%)	431 (79.2%)	
**Total**	566 (100%)	22 (100%)	544 (100%)	
**Exposure/Infection**				
**Single**	136 (21.69%)	27 (62.8%)	109 (18.7%)	**≤0.001**
**Multiple**	491 (78.31%)	16 (37.2%)	475 (81.3%)	
**Total**	627 (100%)	41 (100%)	584 (100%)	

Table S3. Comparison of results by diagnostic methods and serology.

Species	Diagnostic method	Diagnostic method result	Serology -	Serology +
P. falciparum	qPCR	-	64	580
		+	0	71
A. lumbricoides	Microscopy	-	541	141
		+	26	7
	qPCR	-	517	126
		+	36	11
	Microscopy and/or PCR	-	525	137
		+	42	11
S. stercoralis	Microscopy	-	519	188
		+	1	7
	qPCR	-	489	130
		+	14	57
	Microscopy and/or qPCR	-	505	136
		+	15	59
Hookworm	Microscopy	-	196	401
		+	22	96
	qPCR	-	173	333
		+	38	146
	Microscopy and/or PCR	-	175	339
		+	43	158
T. trichiura	Microscopy	-	318	342
		+	20	35
	qPCR	-	283	313
		+	45	49
	Microscopy and/or qPCR	-	289	323
		+	49	54
Schistosoma spp.	Microscopy	-	393	290
		+	8	24
	qPCR	-	371	248
		+	19	52
	Microscopy and/or qPCR	-	376	253
		+	25	61

Gain in positive results by qPCR and/or microscopy in dark grey. Gain in positive results by serology in light grey.

Table S4. Classification of locations based on percentage of individuals infected with *Plasmodium falciparum* or helminths.

Location	MAL-	MAL+	Total	MAL + (%)	Median % of infection	Q1	Q3	Classification with respect to the median % of infection
3 Fevereiro	101	15	116	12.931	13.203	6.823	21.569	Low
Calanga	122	19	141	13.475				High
Ilha Josina	22	8	30	26.667				High
Maluana	179	9	188	4.787				Low
Manhiça-sede	180	9	189	4.762				Low
Xinavane	40	11	51	21.569				High
	**HEL-**	**HEL+**		**HEL + (%)**				
3 Fevereiro	51	65	116	56.034	48.814	45.331	51.596	High
Calanga	36	105	141	74.468				High
Ilha Josina	24	6	30	20				Low
Maluana	91	97	188	51.596				High
Manhiça-sede	102	87	189	46.032				Low
Xinavane	28	23	51	45.098				Low

Malaria (MAL) and helminth (HEL) percentage of infection in each location of the study were calculated and these were classified into “high” and “low” prevalence locations based on higher or lower percentage of infected individuals compared to the median percentage of infections from all locations by type of parasite.

Table S5. Risk of exposure to or infection with *Plasmodium falciparum* or helminths.

		Outcome (Exposure/ infection)			Outcome (Exposure/ infection)		
		MAL +	MAL -	OR [95% CI]	HEL+	HEL-	OR [95% CI]
Predictor (Exposure/ infection)	MAL +				584	64	1.620* [0.858 – 2.992]
	MAL -				43	21	
	HEL+	584	42	1.745** [0.923 – 3.221]			
	HEL-	67	21				

The models were adjusted by covariables selected based on their significant association with the outcome variable in univariable models:

* Log_{10} age and socioeconomic score

** Log_{10} age.

OR: odds ratio, CI: Confidence interval.

SUPPLEMENTARY FIGURES

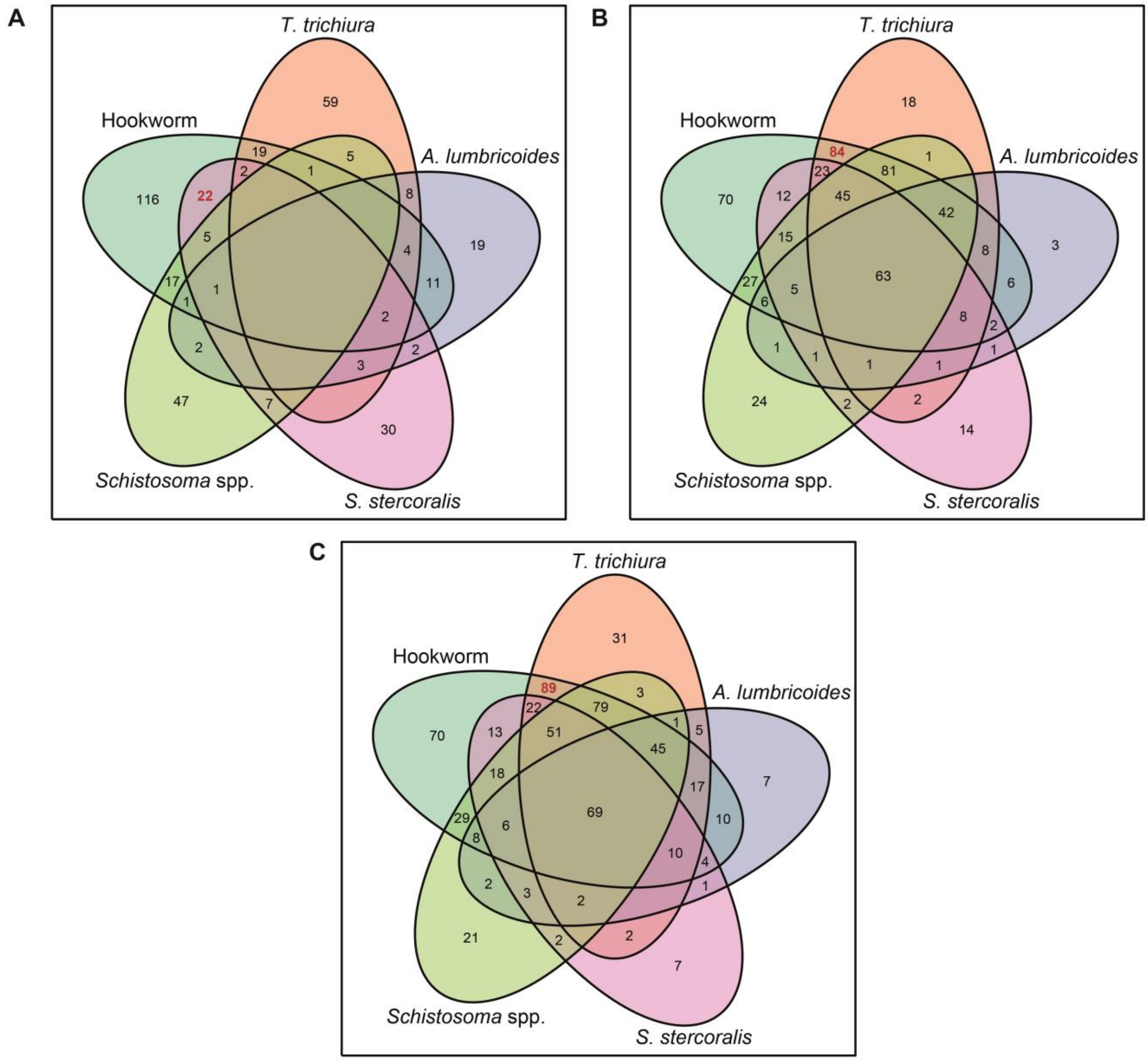

Figure S1. Venn diagrams showing the combinations of (A) coinfections, (B) coexposure and (C) coexposure/coinfection of helminth species. The most common combination of species in each classification is highlighted in red.

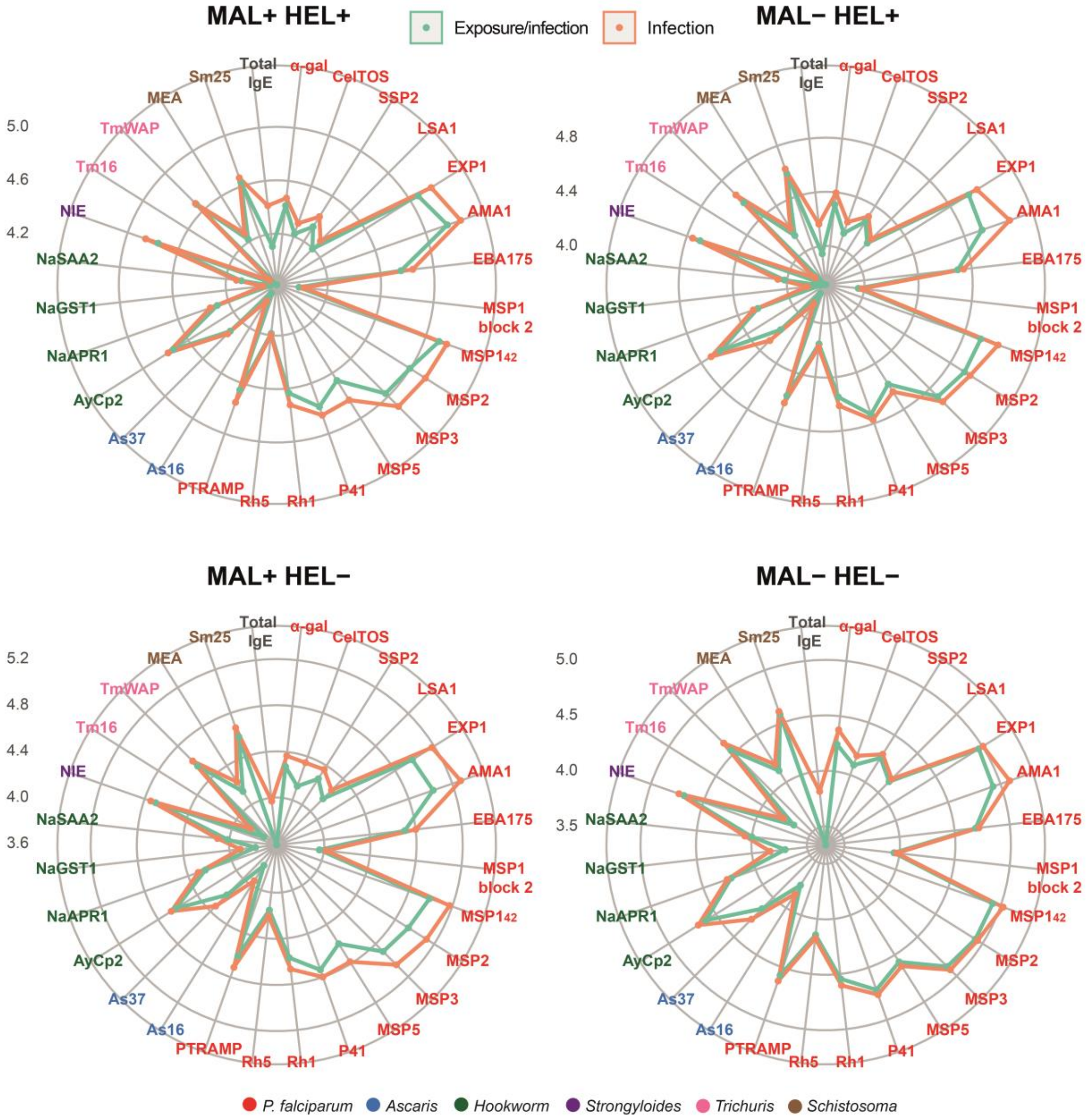

Figure S2. Comparison of antibody responses between groups classified by exposure/infection and only infection. In the vertices, the antibody responses are grouped by specific IgG to *P. falciparum* antigens (α-gal, CelTOS, SSP2, LSA1, EXP1, AMA1, EBA175, MSP1 block 2, MSP1$_{42}$, MSP2, MSP3, MSP5, P41, Rh5, PTRAMP), helminth antigens (As16 and As37 [*Ascaris lumbricoides*], AyCp2, NaAPR1, NaGST1, NaSAA2 [Hookworm], NIE [*Strongyloides stercoralis*], Tm16 and TmWAP [*Trichuris trichiura*], MEA and Sm25

[*Schistosoma* spp.] or total IgE. The colored lines represent the median of antibody levels expressed as log_{10}-transformed median fluorescence levels (MFI). Comparisons were made within study groups: no exposure/infection (green) or no infection (orange) (MAL- HEL-), only exposure/infection (green) or only infection (orange) with helminths (MAL- HEL+), only exposure/infection (green) or only infection (orange) with *P. falciparum* (MAL+ HEL-) and coexposure/coinfection (green) or coinfection (orange) with *P. falciparum* and helminths (MAL+ HEL+).

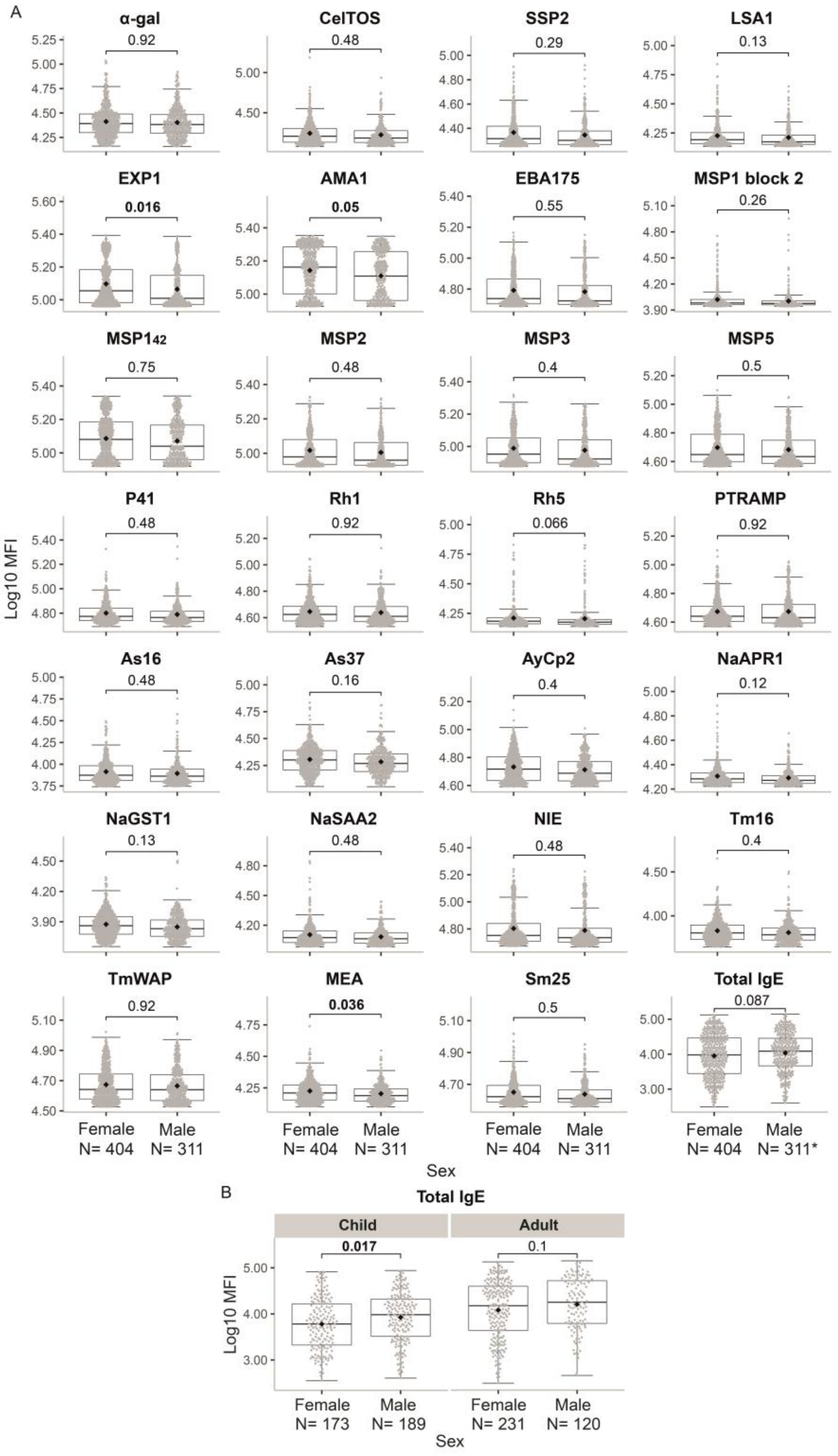

Figure S3. Comparison of antibody responses by sex. (A) Antibody levels expressed as the log_{10}-transformed median fluorescence (MFI) of specific IgG to *P. falciparum* antigens (α-gal, CelTOS, SSP2, LSA1, EXP1, AMA1, EBA175, MSP1 block 2, $MSP1_{42}$, MSP2, MSP3, MSP5, P41,

Rh5, PTRAMP), helminth antigens (As16 and As37 [*Ascaris lumbricoides*], AyCp2, NaAPR1, NaGST1, NaSAA2 [Hookworm], NIE [*Strongyloides stercoralis*], Tm16 and TmWAP [*Trichuris trichiura*], MEA and Sm25 [*Schistosoma* spp.] or total IgE. **(B)** Log_{10}-transformed MFI levels of total IgE stratified by age. The boxplots represent the median (bold line), the mean (black diamond), the 1st and 3rd quartiles (box) and the largest and smallest values within 1.5 times the inter-quartile range (whiskers). Data beyond the end of the whiskers are outliers. Statistical comparison between groups was performed by Wilcoxon rank sum test and the adjusted p-values by the Benjamini-Hochberg approach are shown. Statistically significant p-values are highlighted in bold. *N= 309.

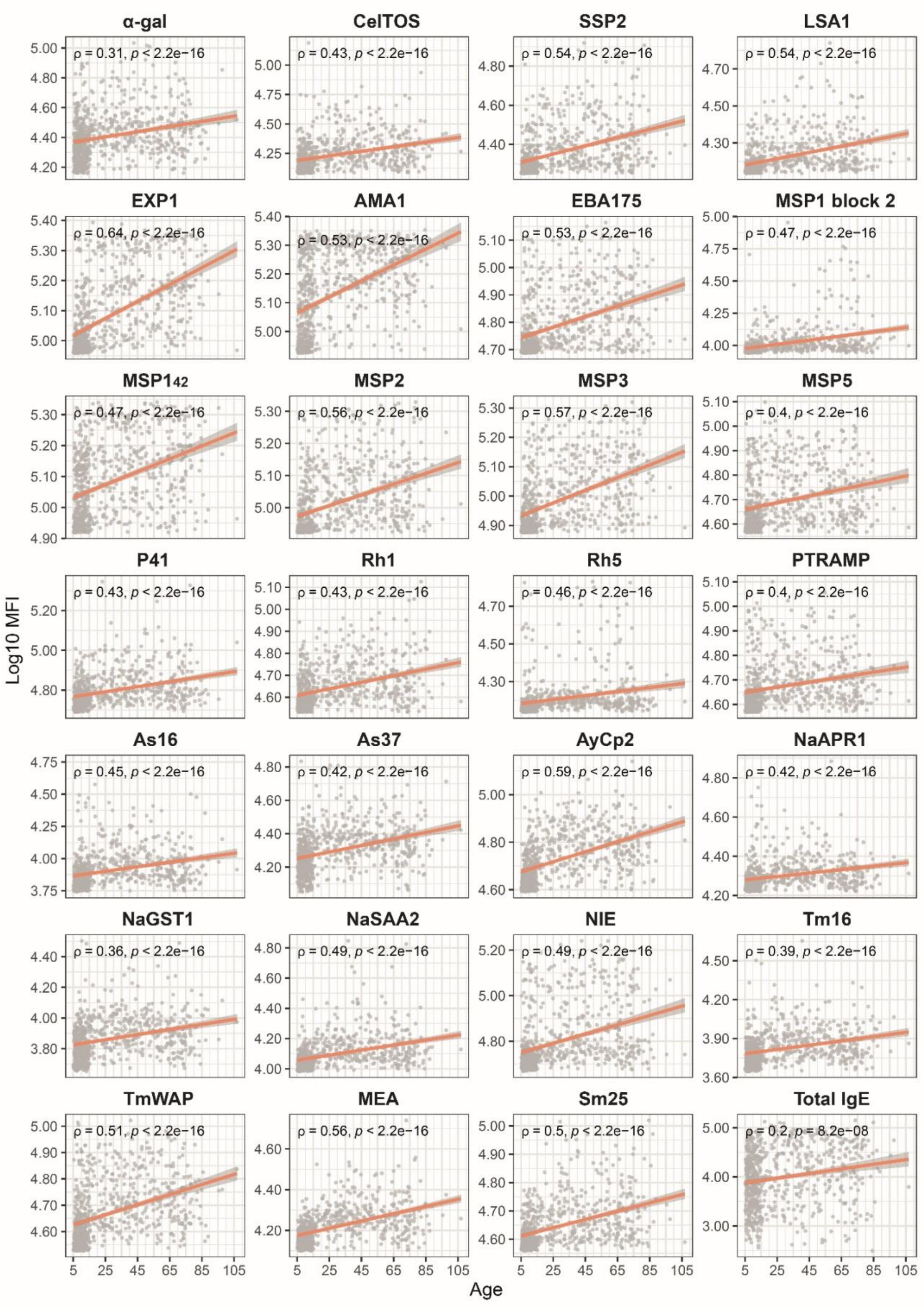

Figure S4. Antibody levels correlation with age. Antibody levels expressed as the $\log_{10}$-transformed median fluorescence (MFI) of specific IgG to *P. falciparum* antigens (α-gal, CelTOS, SSP2, LSA1, EXP1, AMA1, EBA175, MSP1 block 2, $MSP1_{42}$, MSP2, MSP3, MSP5, P41,

Rh5, PTRAMP), helminth antigens (As16 and As37 [*Ascaris lumbricoides*], AyCp2, NaAPR1, NaGST1, NaSAA2 [Hookworm], NIE [*Strongyloides stercoralis*], Tm16 and TmWAP [*Trichuris trichiura*], MEA and Sm25 [*Schistosoma* spp.] or total IgE. Age is expressed in years. ρ (rho) and p-values were calculated by Spearman, shaded areas represent 0.95 confidence intervals.

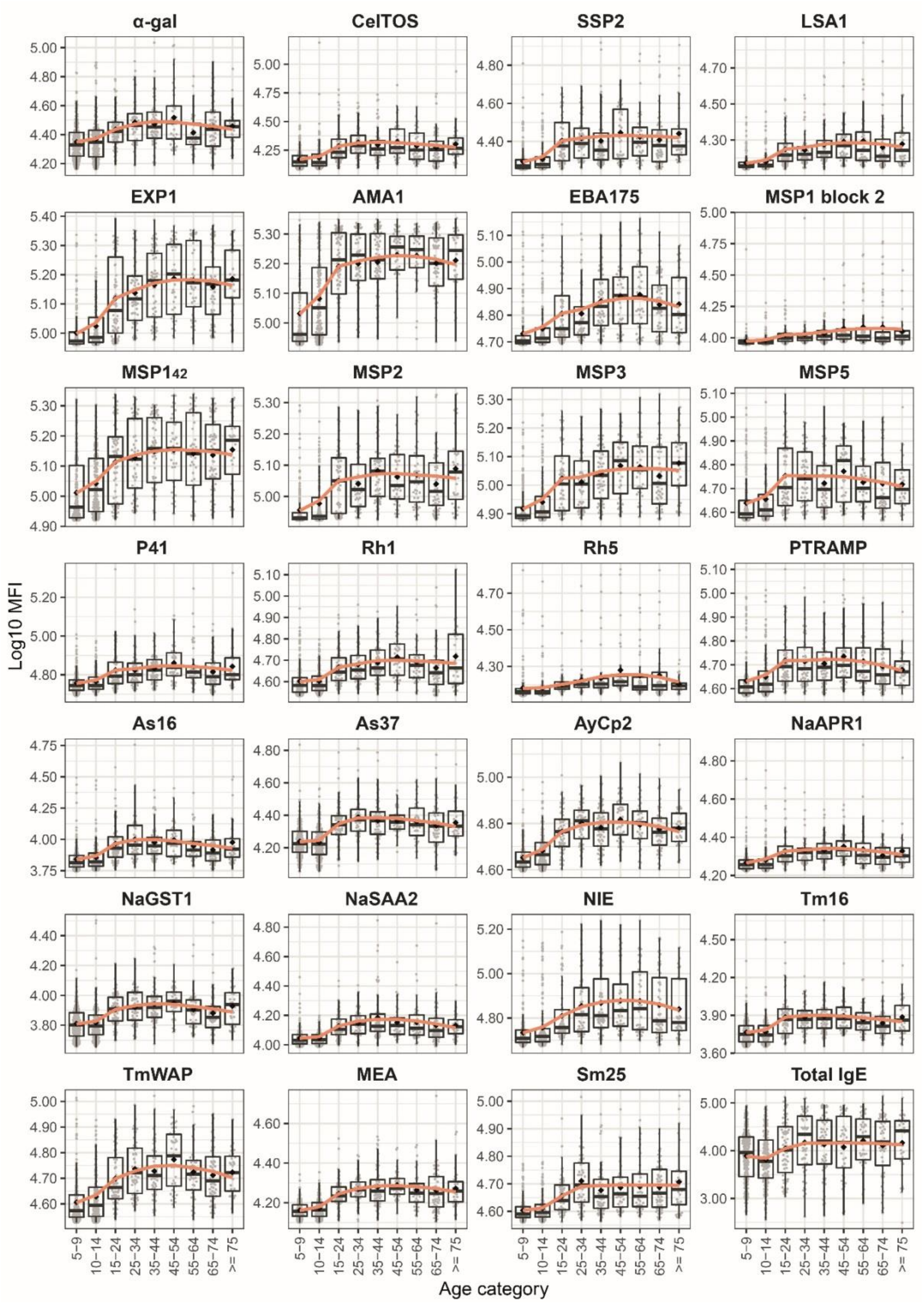

Figure S5. Antibody levels by age groups. Antigen-specific IgG levels against *Plasmodium falciparum* and helminth antigens and total IgE levels are expressed as $\log_{10}$-transformed median fluorescence levels (MFI). Age categories are grouped in 5 years for children and 10 years for adults and the boxplots represent the median (bold line), the mean (black

diamond), the 1st and 3rd quartiles (box) and the largest and smallest values within 1.5 times the inter-quartile range (whiskers). Data beyond the end of the whiskers are outliers. The kinetics curves in orange were calculated using the locally estimated scatterplot smoothing (LOESS) method. *P. falciparum* antigens: α-gal, CelTOS, SSP2, LSA1, EXP1, AMA1, EBA175, MSP1 block 2, $MSP1_{42}$, MSP2, MSP3, MSP5, P41, Rh5, PTRAMP. Helminth antigens: As16 and As37 (*Ascaris lumbricoides*), AyCp2, NaAPR1, NaGST1, NaSAA2 (Hookworm), NIE (*Strongyloides stercoralis*), Tm16 and TmWAP (*Trichuris trichiura*), MEA and Sm25 (*Schistosoma* spp).

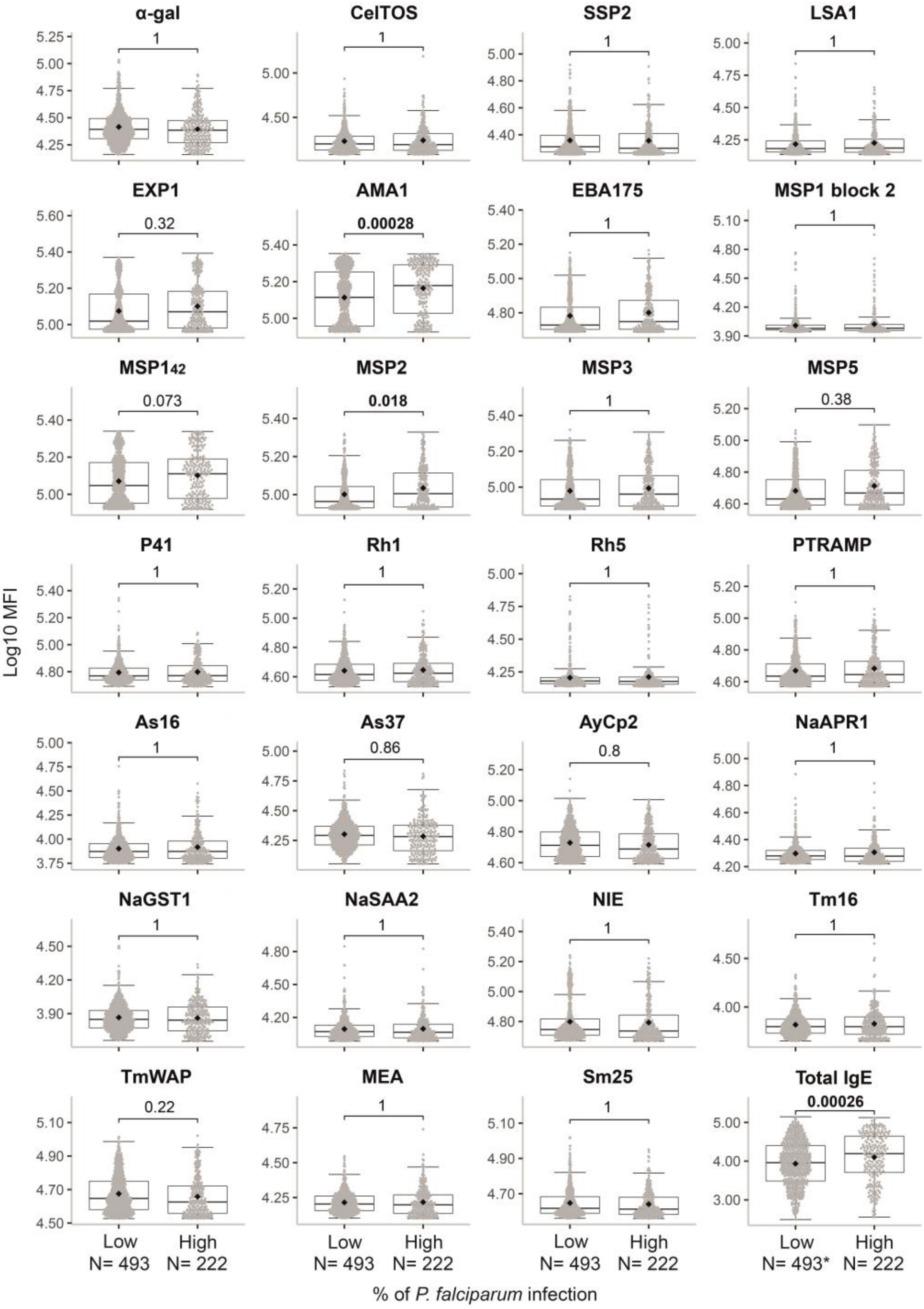

Figure S6. Comparison of antibody responses by percentage of *P. falciparum* infection by location. Antibody levels expressed as the log_{10}-transformed median fluorescence (MFI) of specific IgG to *P. falciparum* antigens (α-gal, CelTOS, SSP2, LSA1, EXP1, AMA1, EBA175, MSP1 block 2, $MSP1_{42}$, MSP2, MSP3, MSP5, P41, Rh5, PTRAMP), helminth antigens (As16

and As37 [*Ascaris lumbricoides*], AyCp2, NaAPR1, NaGST1, NaSAA2 [Hookworm], NIE [*Strongyloides stercoralis*], Tm16 and TmWAP [*Trichuris trichiura*], MEA and Sm25 [*Schistosoma* spp.] or total IgE. In the X-axis, locations were classified into “high” and “low” prevalence based on whether the percentage of *P. falciparum* infection was lower or higher than the median percentage of *P. falciparum* infections in all locations. The boxplots represent the median (bold line), the mean (black diamond), the 1st and 3rd quartiles (box) and the largest and smallest values within 1.5 times the inter-quartile range (whiskers). Data beyond the end of the whiskers are outliers. Statistical comparison between groups was performed by Wilcoxon rank sum test and the adjusted p-values by the Benjamini-Hochberg approach are shown. Statistically significant p-values are highlighted in bold. *N= 491.

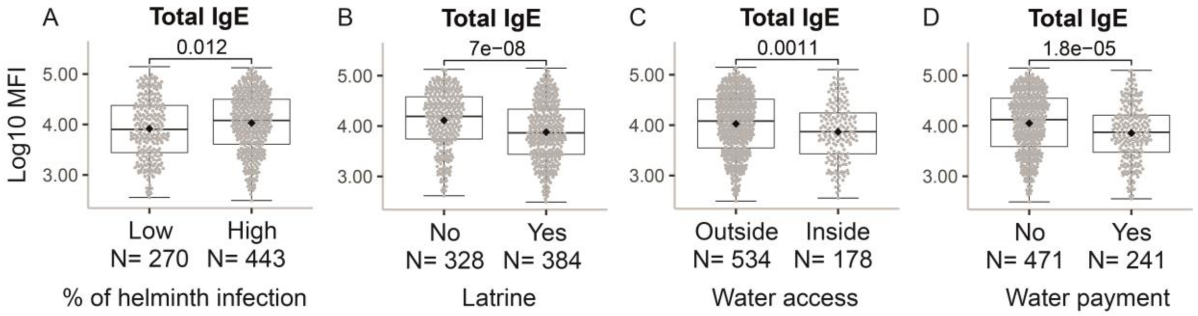

Figure S7. Comparison of total IgE antibody responses by different variables. (A) percentage of helminth infection by location classified into “high” and “low” prevalence based on whether the percentage of helminth infection was lower or higher than the median percentage of helminth infections in all locations; **(B)** ownership of latrine; **(C)** accessibility to piped water in the household; and **(D)** payment of piped water. Antibody levels expressed as the $\log_{10}$-transformed median fluorescence (MFI) of total IgE. The boxplots represent the median (bold line), the mean (black diamond), the 1st and 3rd quartiles (box) and the largest and smallest values within 1.5 times the inter-quartile range (whiskers). Data beyond the end of the whiskers are outliers. Statistical comparison between groups was performed by Wilcoxon rank sum test and adjusted p-values by the Benjamini-Hochberg approach are shown.

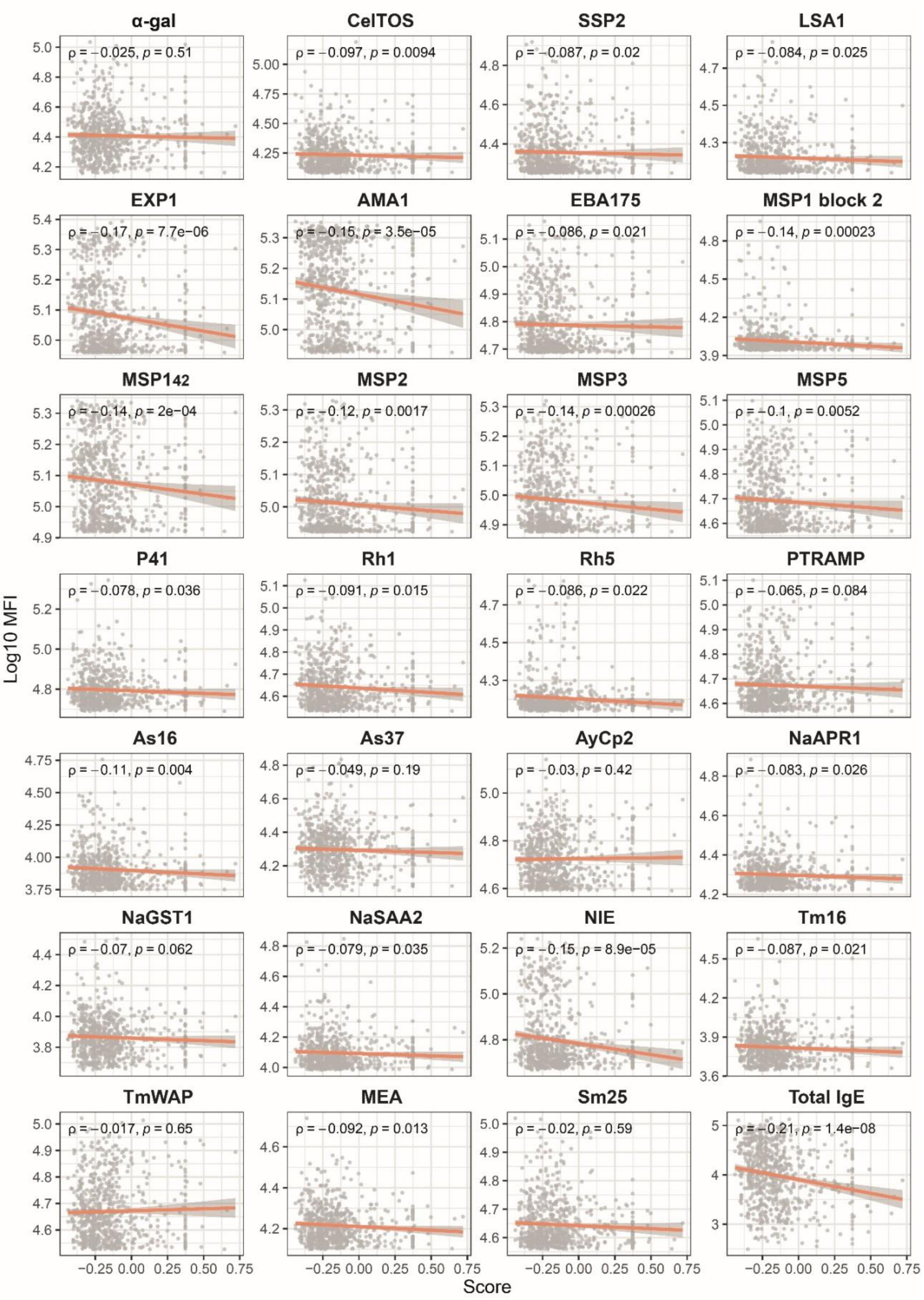

Figure S8. Antibody levels correlation with socioeconomic score. Antibody levels expressed as the log_{10}-transformed median fluorescence (MFI) of specific IgG to *P. falciparum* antigens (α-gal, CelTOS, SSP2, LSA1, EXP1, AMA1, EBA175, MSP1 block 2, $MSP1_{42}$, MSP2, MSP3,

MSP5, P41, Rh5, PTRAMP), helminth antigens (As16 and As37 [*Ascaris lumbricoides*], AyCp2, NaAPR1, NaGST1, NaSAA2 [Hookworm], NIE [*Strongyloides stercoralis*], Tm16 and TmWAP [*Trichuris trichiura*], MEA and Sm25 [*Schistosoma* spp.] or total IgE. ρ (rho) and p-values were calculated by Spearman, shaded areas represent 0.95 confidence intervals.

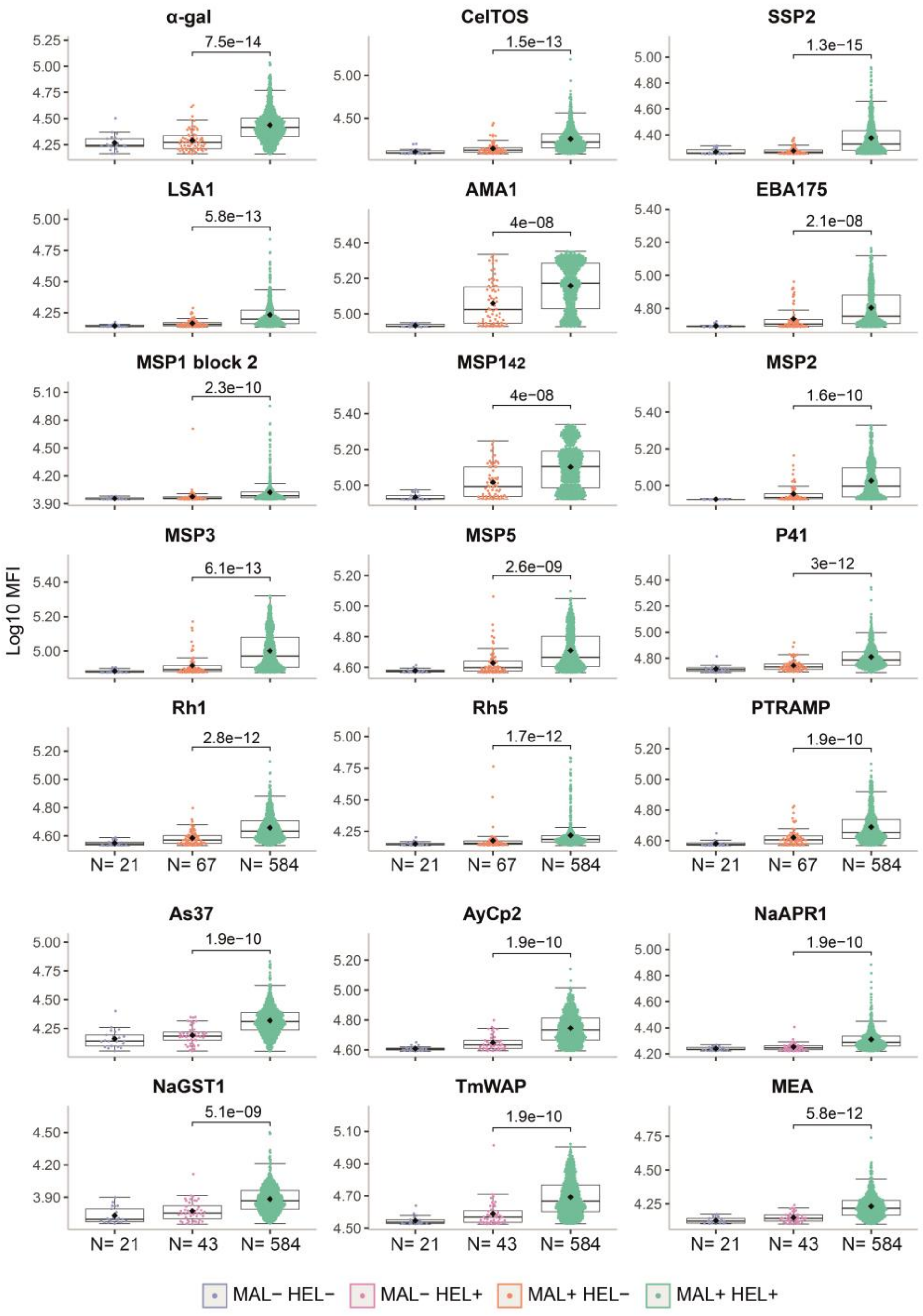

Figure S9. Comparison of antibody responses between exposure/infection groups.

Antibody levels expressed as the $\log_{10}$-transformed median fluorescence (MFI) of specific IgG to *P. falciparum* antigens (α-gal, CelTOS, SSP2, LSA1, AMA1, EBA175, MSP1 block 2, $MSP1_{42}$, MSP2, MSP3, MSP5, P41, Rh5, PTRAMP), helminth antigens (As37 [*Ascaris lumbricoides*], AyCp2, NaAPR1, NaGST1 [Hookworm], TmWAP [*Trichuris trichiura*], Sm25 [*Schistosoma* spp.]. The boxplots represent the median (bold line), the mean (black

diamond), the 1st and 3rd quartiles (box) and the largest and smallest values within 1.5 times the inter-quartile range (whiskers). Data beyond the end of the whiskers are outliers. Statistical comparison between groups was performed by Wilcoxon rank sum test and adjusted p-values by the Benjamini-Hochberg approach are shown. Study groups are shown in colors: no exposure/infection (violet) (MAL- HEL-), only exposure/infection with helminths (pink) (MAL- HEL+), only exposure/infection with *P. falciparum* (orange) (MAL+ HEL-) and coexposure/coinfection with *P. falciparum* and helminths (green) (MAL+ HEL+).

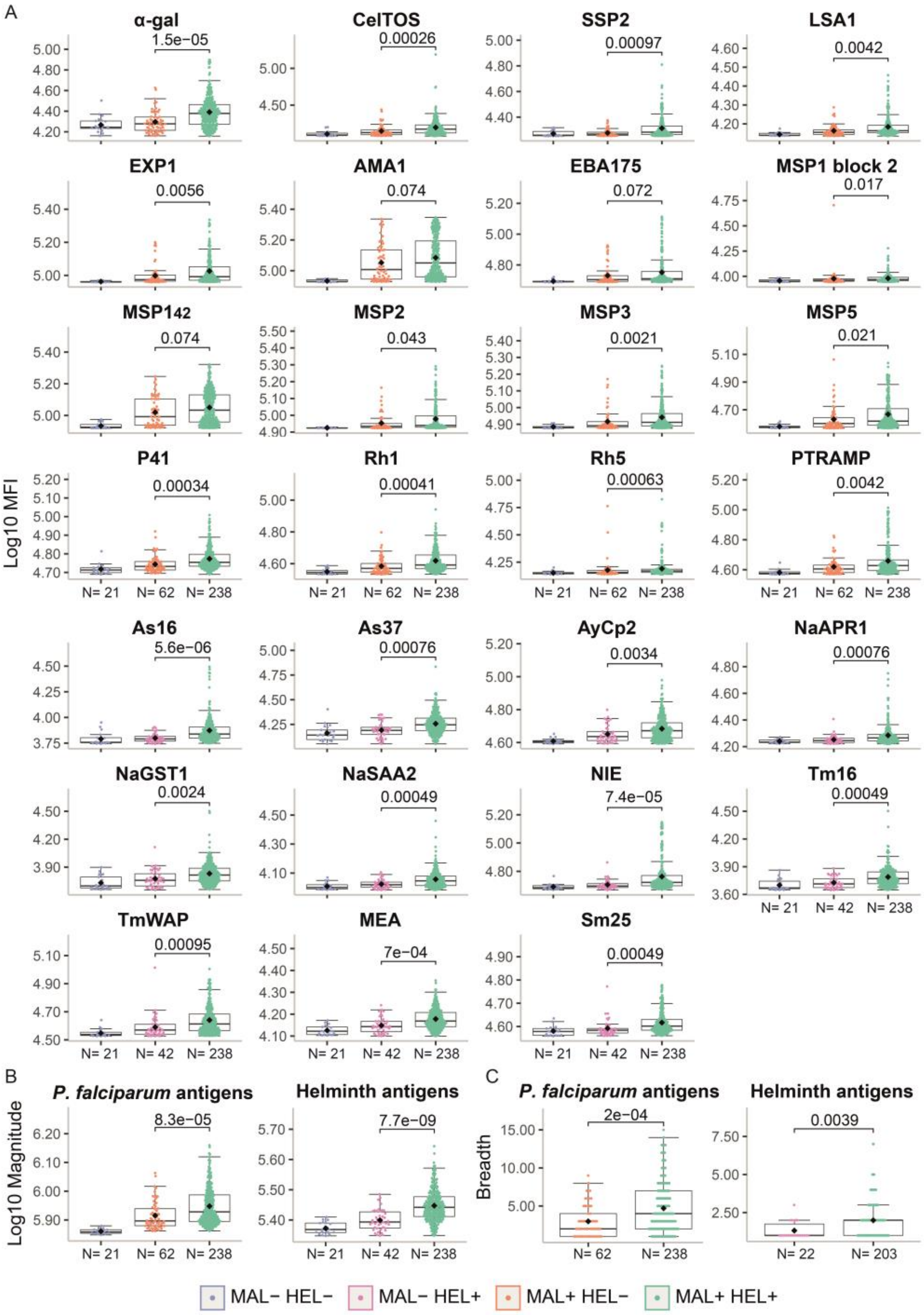

Figure S10. Comparison of antibody responses between exposure/infection groups in children. Antibody responses are expressed as **(A)** the log_{10}-transformed median fluorescence (MFI) of specific IgG to *P. falciparum* antigens (α-gal, CelTOS, SSP2, LSA1, EXP1, AMA1, EBA175, MSP1 block 2, $MSP1_{42}$, MSP2, MSP3, MSP5, P41, Rh5, PTRAMP), helminth antigens (As16 and As37 [*Ascaris lumbricoides*], AyCp2, NaAPR1, NaGST1, NaSAA2 [Hookworm], NIE [*Strongyloides stercoralis*], Tm16 and TmWAP [*Trichuris trichiura*], MEA

and Sm25 [*Schistosoma* spp.] or total IgE, **(B)** the $\log_{10}$-transformed magnitude of response (sum of MFI), and **(C)** the breadth of response (number of seropositive antigen-specific IgG responses). The boxplots represent the median (bold line), the mean (black diamond), the 1st and 3rd quartiles (box) and the largest and smallest values within 1.5 times the inter-quartile range (whiskers). Data beyond the end of the whiskers are outliers. Statistical comparison between groups was performed by Wilcoxon rank sum test and the exact p-values are shown. Adjusted p-values by the Benjamini-Hochberg approach are shown in brackets. Study groups are shown in colors: no exposure/infection (violet) (MAL- HEL-), only exposure/infection with helminths (pink) (MAL- HEL+), only exposure/infection with *P. falciparum* (orange) (MAL+ HEL-) and coexposure/coinfection with *P. falciparum* and helminths (green) (MAL+ HEL+).

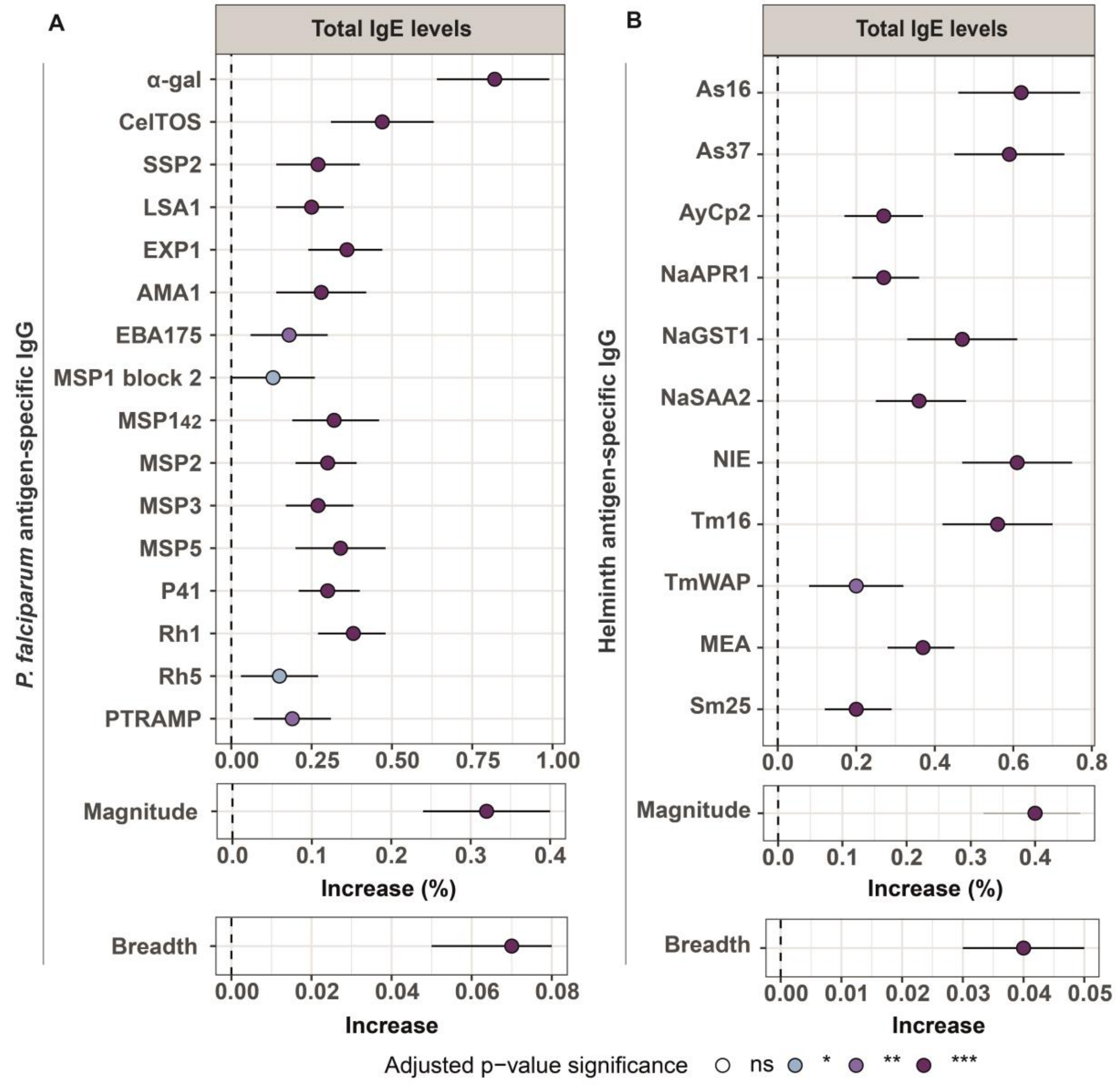

Figure S11. Association of total IgE levels with antibody levels in multivariable linear regression models. Forest plots show the association of total IgE levels with **(A)** *P. falciparum* antigens, magnitude and breadth of response or **(B)** helminth antigens, magnitude (sum of antigen-specific IgG levels) and breadth (number of seropositive antigen-specific IgG responses) of response. Multivariable linear regression models were fitted to calculate the estimates (dots) and 95% confidence intervals (CI) (lines). The represented values are the transformed betas and CI showing the percentage increases for each 10% increase in total IgE levels (log-log models) for antigen-specific IgG levels and magnitude of

response (log-log models). For breadth of response (linear-log models), the betas and 95% CI were transformed to represent the additive effect on the breadth of response of a 10% increase in total IgE levels (see “Statistical analysis” section for more details). The color of the dots represents the p-value significance after adjustment for multiple testing by Benjamini-Hochberg, where ns= not significant, * = p-value ≤ 0.05, ** = p-value ≤ 0.01 and *** = p-value ≤ 0.001. Models were adjusted by age and percentage of *P. falciparum* infection by location for *P. falciparum* antigens and age for helminth antigens.

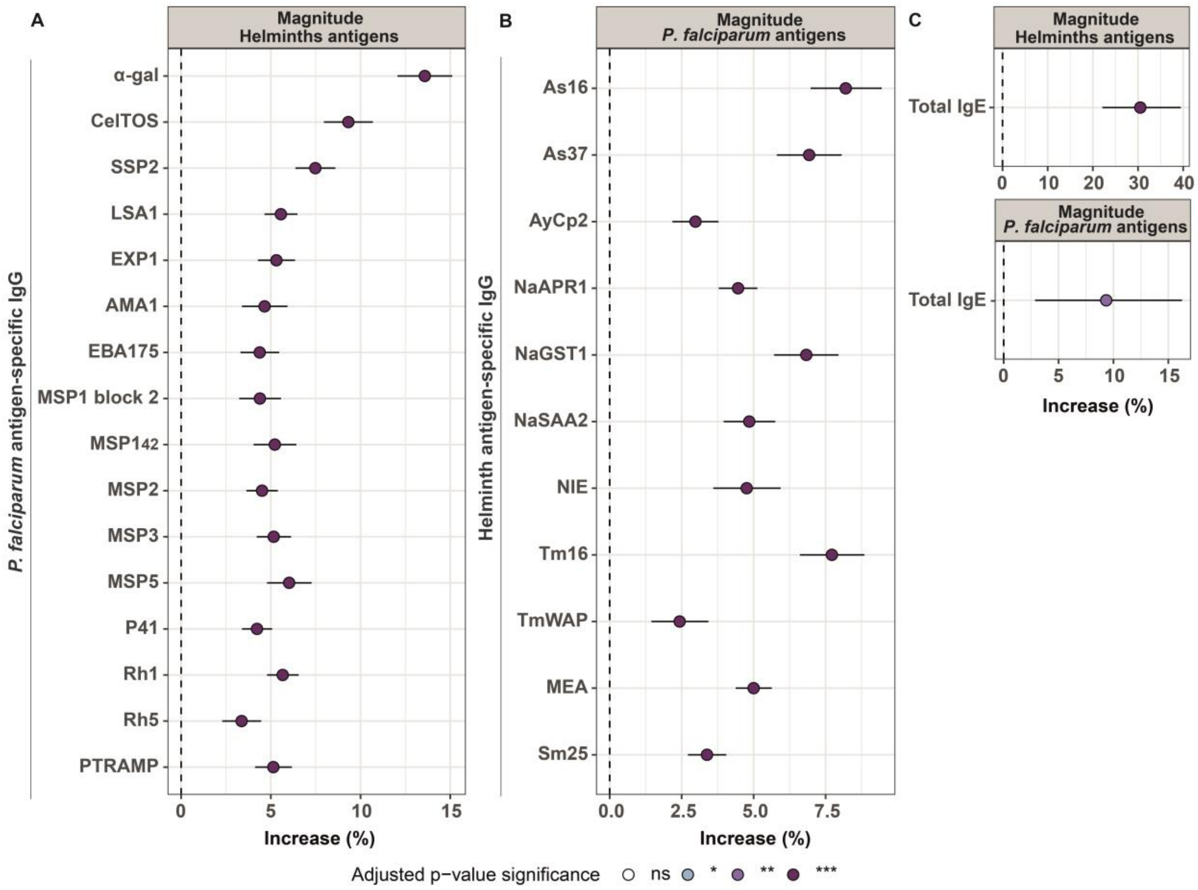

Figure S12. Association of magnitude of response with antibody levels in multivariable linear regression models. Forest plots show the association of **(A)** magnitude of response to helminth antigens with *P. falciparum* antigens, **(B)** magnitude of response to *P. falciparum* antigens with helminth antigens and **(C)** magnitude of response to helminth and *P. falciparum* antigens with total IgE levels. The magnitude of response was calculated as the sum of all specific IgG levels (MFI) to the different antigens belonging to *P. falciparum* or helminths. Multivariable linear regression models were fitted to calculate the estimates (dots) and 95% confidence intervals (CI) (lines). The represented values are the transformed betas and CI showing the percentage increase for each 10% increase in the magnitude of response (log-log models) (see "Statistical analysis" section for more details). The color of the dots represents the p-value significance after adjustment for multiple testing by Benjamini-Hochberg, where ns= not significant, * = p-value ≤ 0.05, ** = p-value ≤ 0.01 and

*** = p-value ≤ 0.001. Models were adjusted by age and percentage of *P. falciparum* infection by location for *P. falciparum* antigens, age for helminth antigens and sex, percentage of helminth infection by location, ownership of latrine, piped water accessibility, payment for piped water and socioeconomic score for total IgE.

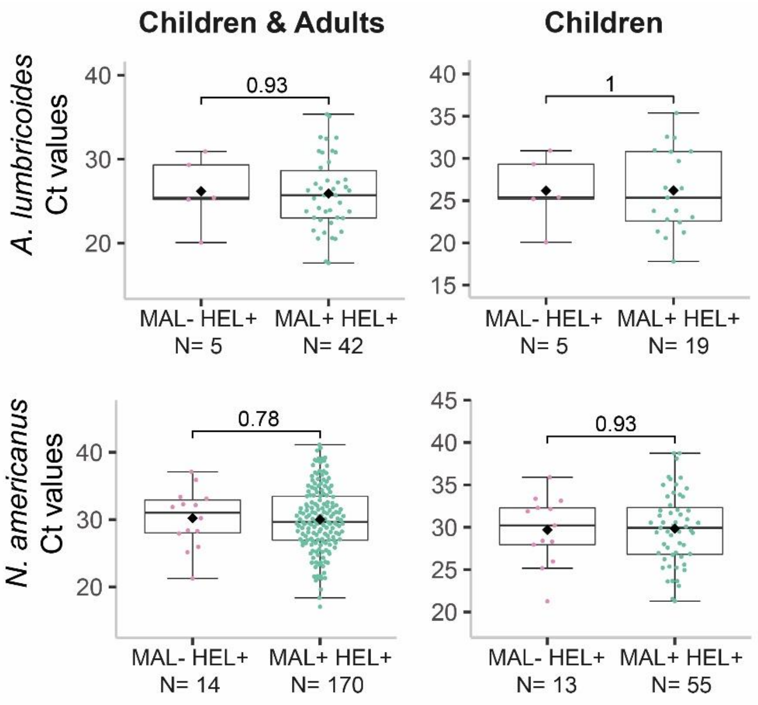

Figure S13. Parasite burden by infection groups. The levels of parasite burden are represented as the Ct values and are compared between the coexposure/coinfection group (MAL+ HEL+) and the single helminth exposure/infection group (MAL- HEL+) for *Ascaris lumbricoides and Necator americanus*. For each parasite, data for all individuals with parasite burden information or only children are displayed. The boxplots represent the median (bold line), the mean (black diamond), the 1st and 3rd quartiles (box) and the largest and smallest values within 1.5 times the inter-quartile range (whiskers). Data beyond the end of the whiskers are outliers. Statistical comparison between groups was performed by Wilcoxon rank sum test and the exact p-values are shown.

SUPPLEMENTARY METHODS

Data pre-processing

For antigen-specific IgG quantification results, we selected the dilution that was within the quantitative range of the PC curve, ideally in the linear part of it. Briefly, the point of the PC curve with the maximum slope was calculated per plate and antigen. The dilution of the sample with the MFI value more similar to this maximum slope point was chosen, as the maximum slope is the most reliable part of the PC curve for quantification. The selection of the dilution was performed after checking for hook effect, since it could disturb the results.

Then, we performed a double normalization to correct a batch effect caused by the use of two different streptavidin-R-phycoerythrin lots. The first normalization was intra-batch to correct for technical artefacts between plates within the same streptavidin-R-phycoerythrin lot. The second normalization was inter-batch to correct for the differences due to the different lots of streptavidin-R-phycoerythrin. First, MFI values after the selection of sample dilution were normalized by multiplying each MFI by the first normalization factor estimated by plate and antigen within each batch. To do so, first an average curve per antigen and batch was calculated by performing the mean of each of the dilution points of the PC curves (i.e.: mean of dilution 1 of all plates within batch 1 for AMA1). Next, the dilution point closest to the maximum slope of the average curve per antigen and batch was identified and denominated as the “normalization dilution point”. This normalization dilution point was then used to calculate the first normalization factor, which was the ratio of the normalization dilution point of each plate curve between the normalization point of the average curve per antigen, plate, and batch. The second normalization factor was obtained by calculating the ratio of the normalization dilution point per antigen between the two

batches. Given that the batch 1 included 15 plates and the batch 2 only 6, the reference was batch 1 because its sample size was bigger. Therefore, the second normalization factor was applied to the batch 2 by multiplying the MFI values by the second normalization factor. In this step, the hook effect was also considered. Finally, a correction for the dilution factor was applied to be able to compare samples tested at different dilutions for IgG. The correction factor was obtained with a regression method based on the maximum slope of the batch 1 average curve for each antigen. The regression line has the next formula: $\boldsymbol{y}=\boldsymbol{mx}+\boldsymbol{b}$, where $\boldsymbol{y}$ is the final corrected MFI value, $\boldsymbol{m}$ is the maximum slope, $\boldsymbol{x}$ the log10-transformed dilution factor and $\boldsymbol{b}$ the normalized MFI.

For total IgE, neither selection of dilution, nor a correction by dilution was needed for the data pre-processing. However, normalization was also required due to the batch effect caused by the different streptavidin-R-phycoerythrin lots. MFI values were normalized by calculating the normalization factor of each plate. This factor was obtained by calculating the ratio of the MFI values from the elution control (EC) 1 per plate between the average of all plates. The EC 1 was selected for normalization since the performance of the PC curve was not good enough to use as a reference. Among the ECs, EC 1 had the lowest CV% (EC1 = 6.28%, EC2 = 23.47% and EC3 = 20.86%).

Data from blanks were excluded since they were negligible.

Table S6. Antigens included in the multiplex panel

Parasite	Antigen	Life stage	Abbreviation and/or information	Rationale	Ref.
**STH**					
Necator americanus	NaGST1	A	Glutathione S-transferase involved in haemoglobin digestion	Candidate vaccine antigen	(1)
Necator americanus	NaAPR1	A	Aspartic protease involved in haemoglobin digestion	Candidate vaccine antigen	(1)
Necator americanus	NaSAA2	L	Surface associated antigen	Candidate vaccine antigen	(2)
Ancylostoma ceylanicum	AyCp2	A	Cysteine protease involved in haemoglobin digestion	Candidate vaccine antigen	(3)
Trichuris muris	TmWAP	A	Whey acidic protein, secreted immunodominant protein	Candidate vaccine antigen	(4)
Trichuris muris	Tm16	A	Secreted immunodominant protein	Candidate vaccine antigen	(5)
Ascaris suum	As16	L / A	Secreted immunodominant protein	Candidate vaccine antigen	(6)
Ascaris suum	As37	L / A	Secreted immunodominant protein	Candidate vaccine antigen	(7)
Strongyloides stercoralis	NIE	L / A	Recombinant antigen used for immunodiagnostic	Exposure to larvae infection	(8)
Schistosoma					
Schistosoma spp.	MEA	Egg	Major component of schistosome eggs	Immunogenic antigen	(9)
Schistosoma mansoni	Sm25	A	Tegumental glycoprotein	Involved in protection	(10)
Plasmodium falciparum	α-gal	PE	Alpha-galactosidase also expressed in bacteria	Involved in malaria protection	(11, 12)
	CelTOS	PE	Cell-traversal protein	Exposure to SPZ infection	(13, 14)
	SSP2(TRAP)	PE	Thrombospondin adhesive protein	Exposure to SPZ infection	(15, 16)
*The antigen fragments and variants included	LSA1	LS	Liver stage antigen	Expressed in infected hepatocytes	(17, 18)
	EXP1	LS/BS	Exported protein	Exposure to asexual BS	(19)

are: AMA1 FVO, EBA175 reg2 PfF2, MSP1 Block 2 MAD20, $MSP1_{42}$ 3D7, MSP2 full length CH150 and MSP3 3D7	AMA1*	BS	Apical membrane antigen	Exposure to asexual BS	(20)
	EBA175*	BS	Erythrocyte binding antigen	Exposure to asexual BS	(21)
	MSP1 bl 2*	BS	Merozoite surface protein	Exposure to asexual BS	(22)
	$MSP1_{42}$*	BS	Merozoite surface protein	Exposure to asexual BS	(23)
	MSP2*	BS	Merozoite surface protein	Exposure to asexual BS	(24)
	MSP3*	BS	Merozoite surface protein	Exposure to asexual BS	(25)
	MSP5	BS	Merozoite surface protein	Exposure to asexual BS	(26)
	P41	BS	Unknown function	Exposure to asexual BS	(27)
	Rh1	BS	Reticulocyte binding protein	Exposure to asexual BS	(28)
	Rh5	BS	Reticulocyte binding protein	Exposure to asexual BS	(29)
	PTRAMP	BS	Thrombospondin apical protein	Exposure to asexual BS	(30)
Others	GST		Glutathione S-transferase	Control fusion protein	

STH: Soil-transmitted helminths, A: adult, L: larvae, PE: pre-erythrocytic stage, LS: liver-stage, BS: blood-stage, SPZ: sporozoite, Ref.: References

Table S7. Panel of antigens with their corresponding optimal coupling conditions

Antigen	Coupling concentration	Buffer
NaGST1	100 µg/ml	MES
NaAPR1	30 µg/ml	MES
NaSAA2	100 µg/ml	MES
AyCp2	50 µg/ml	MES
TmWAP	30 µg/ml	MES
Tm16	50 µg/ml	MES
As16	50 µg/ml	MES
As37	75 µg/ml	MES
NIE	50 µg/ml	MES
MEA	30 µg/ml	MES
Sm25	10 µg/ml	MES
α-gal	30 µg/ml	MES
CelTOS	50 µg/ml	PBS
SSP2 or TRAP	30 µg/ml	PBS
LSA1	30 µg/ml	PBS
EXP1	30 µg/ml	MES
AMA1 FVO	30 µg/ml	MES
EBA175 reg2 PfF2	30 µg/ml	MES
MSP1 Block2 MAD20	50 µg/ml	MES
MSP1 42 3D7	30 µg/ml	MES
MSP2 full length CH150	30 µg/ml	MES
MSP3 3D7	30 µg/ml	MES
MSP5	30 µg/ml	MES
P41	50 µg/ml	MES
PfRh1	30 µg/ml	MES
PfRh5	30 µg/ml	MES
PTRAMP	30 µg/ml	MES
GST	50 µg/ml	MES
anti-IgE	25 µg/ml	MES

MES: Morpholineethane sulfonic acid

SUPPLEMENTARY REFERENCES

1. Adegnika AA et al. Safety and immunogenicity of co-administered hookworm vaccine candidates Na-GST-1 and Na-APR-1 in Gabonese adults: a randomised, controlled, double-blind, phase 1 dose-escalation trial. *Lancet. Infect. Dis.* 2021;21(2):275–285.

2. Asojo OA et al. Crystallization and preliminary X-ray analysis of Na-SAA-2 from the human hookworm parasite Necator americanus. *Acta Crystallogr. Sect. F, Struct. Biol. Cryst. Commun.* 2010;66(Pt 2):172–176.

3. Wei J et al. The hookworm Ancylostoma ceylanicum intestinal transcriptome provides a platform for selecting drug and vaccine candidates. *Parasit. Vectors* 2016;9(1):518.

4. Briggs N et al. Trichuris muris whey acidic protein induces type 2 protective immunity against whipworm. *PLoS Pathog.* 2018;14(8):e1007273.

5. Liu Z et al. Identification, Characterization, and Structure of Tm16 from Trichuris muris. *J. Parasitol. Res.* 2017;2017:4342789.

6. Wei J et al. Yeast-expressed recombinant As16 protects mice against Ascaris suum infection through induction of a Th2-skewed immune response. *PLoS Negl. Trop. Dis.* 2017;11(7):e0005769.

7. Versteeg L et al. Protective immunity elicited by the nematode-conserved As37 recombinant protein against Ascaris suum infection. *PLoS Negl. Trop. Dis.* 2020;14(2):e0008057.

8. Rascoe LN et al. Development of Ss-NIE-1 recombinant antigen based assays for immunodiagnosis of strongyloidiasis. *PLoS Negl. Trop. Dis.* 2015;9(4):e0003694.

9. Silva-Moraes V et al. Serological proteomic screening and evaluation of a recombinant egg antigen for the diagnosis of low-intensity Schistosoma mansoni infections in endemic area in Brazil. *PLoS Negl. Trop. Dis.* 2019;13(3):e0006974.

10. Petzke MM et al. Schistosoma mansoni gene GP22 encodes the tegumental antigen sm25: (1) antibodies to a predicted B-cell epitope of Sm25 cross-react with other candidate vaccine worm antigens; (2) characterization of a recombinant product containing tandem-repeats of thi. *Parasite Immunol.* 2000;22(8):381–395.

11. Yilmaz B et al. Gut microbiota elicits a protective immune response against malaria transmission. *Cell* 2014;159(6):1277–1289.

12. Aguilar R et al. Antibody responses to α-Gal in African children vary with age and site and are associated with malaria protection. *Sci. Rep.* 2018;8(1):9999.

13. Bergmann-Leitner ES et al. Self-adjuvanting bacterial vectors expressing pre-erythrocytic antigens induce sterile protection against malaria. *Front. Immunol.* 2013;4:176.

14. Kusi KA et al. Anti-sporozoite antibodies as alternative markers for malaria transmission intensity estimation. *Malar. J.* 2014;13:103.

15. Khusmith S et al. Protection against malaria by vaccination with sporozoite surface protein 2 plus CS protein. *Science* 1991;252(5006):715–718.

16. Robson KJ et al. A highly conserved amino-acid sequence in thrombospondin, properdin and in proteins from sporozoites and blood stages of a human malaria parasite. *Nature* 1988;335(6185):79–82.

17. Guerin-Marchand C et al. A liver-stage-specific antigen of Plasmodium falciparum

characterized by gene cloning. *Nature* 1987;329(6135):164–167.

18. Zhu J, Hollingdale MR. Structure of Plasmodium falciparum liver stage antigen-1. *Mol. Biochem. Parasitol.* 1991;48(2):223–226.

19. Doolan DL et al. Identification and characterization of the protective hepatocyte erythrocyte protein 17 kDa gene of Plasmodium yoelii, homolog of Plasmodium falciparum exported protein 1. *J. Biol. Chem.* 1996;271(30):17861–17868.

20. Kocken CHM et al. High-level expression of the malaria blood-stage vaccine candidate Plasmodium falciparum apical membrane antigen 1 and induction of antibodies that inhibit erythrocyte invasion. *Infect. Immun.* 2002;70(8):4471–4476.

21. Sim BK, Chitnis CE, Wasniowska K, Hadley TJ, Miller LH. Receptor and ligand domains for invasion of erythrocytes by Plasmodium falciparum. *Science* 1994;264(5167):1941–1944.

22. Cavanagh DR, McBride JS. Antigenicity of recombinant proteins derived from Plasmodium falciparum merozoite surface protein 1. *Mol. Biochem. Parasitol.* 1997;85(2):197–211.

23. Angov E et al. Development and pre-clinical analysis of a Plasmodium falciparum Merozoite Surface Protein-1(42) malaria vaccine. *Mol. Biochem. Parasitol.* 2003;128(2):195–204.

24. Metzger WG et al. Serum IgG3 to the Plasmodium falciparum merozoite surface protein 2 is strongly associated with a reduced prospective risk of malaria. *Parasite Immunol.* 2003;25(6):307–312.

25. Imam M, Singh S, Kaushik NK, Chauhan VS. Plasmodium falciparum merozoite surface

protein 3: oligomerization, self-assembly, and heme complex formation. *J. Biol. Chem.* 2014;289(7):3856–3868.

26. Black CG, Barnwell JW, Huber CS, Galinski MR, Coppel RL. The Plasmodium vivax homologues of merozoite surface proteins 4 and 5 from Plasmodium falciparum are expressed at different locations in the merozoite. *Mol. Biochem. Parasitol.* 2002;120(2):215–224.

27. Taechalertpaisarn T et al. Biochemical and functional analysis of two Plasmodium falciparum blood-stage 6-cys proteins: P12 and P41. *PLoS One* 2012;7(7):e41937.

28. Gaur D, Mayer DCG, Miller LH. Parasite ligand-host receptor interactions during invasion of erythrocytes by Plasmodium merozoites. *Int. J. Parasitol.* 2004;34(13–14):1413–1429.

29. Reddy KS et al. Bacterially expressed full-length recombinant Plasmodium falciparum RH5 protein binds erythrocytes and elicits potent strain-transcending parasite-neutralizing antibodies. *Infect. Immun.* 2014;82(1):152–164.

30. Siddiqui FA et al. A thrombospondin structural repeat containing rhoptry protein from Plasmodium falciparum mediates erythrocyte invasion. *Cell. Microbiol.* 2013;15(8):1341–1356.
